# Long-Term Pollution Does Not Inhibit Denitrification and DNRA by Adapted Benthic Microbial Communities

**DOI:** 10.1007/s00248-023-02241-7

**Published:** 2023-05-24

**Authors:** Elias Broman, Mohanad Abdelgadir, Stefano Bonaglia, Sara C. Forsberg, Johan Wikström, Jonas S. Gunnarsson, Francisco J. A. Nascimento, Sara Sjöling

**Affiliations:** 1 Department of Ecology, Environment and Plant Sciences, Stockholm University, 106 91 Stockholm, Sweden; 2 Baltic Sea Centre, Stockholm University, Stockholm, Sweden; 3 Department of Environmental Science, School of Natural Sciences, Technology and Environmental Studies, Södertörn University, 141 89 Huddinge, Sweden; 4 Department of Marine Sciences, Gothenburg University, 413 19 Gothenburg, Sweden

**Keywords:** Baltic Sea, Chlorinated dibenzofurans, Dioxins, Metagenome, Nitrogen cycling, Sediment

## Abstract

Denitrification in sediments is a key microbial process that removes excess fixed nitrogen, while dissimilatory nitrate reduction to ammonium (DNRA) converts nitrate to ammonium. Although microorganisms are responsible for essential nitrogen (N) cycling, it is not yet fully understood how these microbially mediated processes respond to toxic hydrophobic organic compounds (HOCs) and metals. In this study, we sampled long-term polluted sediment from the outer harbor of Oskarshamn (Baltic Sea), measured denitrification and DNRA rates, and analyzed taxonomic structure and N-cycling genes of microbial communities using metagenomics. Results showed that denitrification and DNRA rates were within the range of a national reference site and other unpolluted sites in the Baltic Sea, indicating that long-term pollution did not significantly affect these processes. Furthermore, our results indicate an adaptation to metal pollution by the N-cycling microbial community. These findings suggest that denitrification and DNRA rates are affected more by eutrophication and organic enrichment than by historic pollution of metals and organic contaminants.

**Supplementary Information:**

The online version contains supplementary material available at 10.1007/s00248-023-02241-7.

## Introduction 

Nitrogen loading has increased globally in many coastal seascapes, mainly due to the increase in agricultural food production and use of chemical fertilizers [1]. This has implications for coastal ecosystems resulting in enhanced primary productivity and eutrophication with episodic hypoxia in bottom water and sediments [1, 2]. Particularly the Baltic Sea ecosystem has a history of exposure to eutrophication with bottom water and sediment hypoxia and anoxia [3]. Microbial denitrification is the main route of nitrate (NO_3_^−^) and nitrite (NO_2_^−^) removal in coastal zones [1]. This anaerobic process reduces NO_3_^−^ and NO_2_^−^ into gaseous nitrogen compounds and typically occurs directly below the oxic sediment surface in coastal environments [4]. Bottom water NO_3_^−^ concentrations and nitrification in the oxic sediment surface regulate the availability of NO_3_^−^ for denitrification in the underlying sediment [4]. Through several redox reduction steps, NO_3_^−^ is ultimately reduced to nitrogen gas (N_2_) [5]. Each step is carried out by enzymes encoded by specific genes and this information has been used in various studies to investigate and compare denitrification gene diversity, abundance, or gene expression. For example, the genes *nirS* and *nirK* encode for nitrite reductases (NO_2_^−^ to NO), and *nosZ* encodes for nitrous oxide reductase responsible for the final step in the denitrification pathway (N_2_O to N_2_) [e.g., 6, 7, 8]. While denitrification provides a route for removal of bioavailable nitrogen (N), dissimilatory nitrate reduction to ammonium (DNRA) instead recycles NO_3_^−^ to NH_4_^+^ [5, 6]. This is an important process that internally recycles fixed N via mineralization/release of NH_4_^+^ followed by subsequent nitrification to NO_3_^−^. Compared to denitrification, DNRA thus prevents NO_3_^−^ being lost as N_2_-gas, potentially supporting eutrophication [7]. Studies investigating DNRA target the gene *nrfA* that encodes for a nitrite reductase enzyme used in DNRA [8]. In addition, the gene *nirB* encodes for a nitrate reductase used in DNRA but might also be used in assimilatory nitrate reduction [9]. The key roles of sediment microorganisms in these processes of cycling essential nitrogen [10] between sediment and water [11], together with the prevalent industrial pollution of urbanized coastal areas, call for a better understanding of how and whether the microbial community’s functioning is impacted in industrially polluted coastal sediments.

Both pollution by toxic compounds (e.g., metals and organic contaminants) and nutrient loading of coastal ecosystems have dramatically increased during the last century as a result of industrial processes, land use changes, increased agricultural practices, and the excessive use of fertilizers [2, 12, 13]. Of particular environmental and societal concern is the pollution by toxic metals and persistent hydrophobic organic compounds (HOCs), such as polychlorinated dibenzodioxins (PCDD) and dibenzofurans (PCDF), because of their high toxicity and long-term effect on biota [14, 15]. Industrially emitted metals often reach concentrations in the sediment that have been shown to be toxic to macrofauna but may also select for species with a tolerance to metals [14, 16]. Previous research indicates that at least some microscopic animals (e.g., specific nematodes) can develop tolerance and survive in sediment with persistent organic contaminants and/or toxic metals [17–19]. Tolerance to metals and hydrophobic organic compounds has, however, been shown to be considerably more common among bacteria due to highly diverse bacterial metabolic capacities and rapid genetic and physiological adaptation [20, 21].

There is currently a lack of studies investigating how high concentrations of metals in sediments affect DNRA rates; however, some previous studies have shown that high concentrations of, e.g., Cd, lead (Pb), copper (Cu), and zinc (Zn) in sediment can inhibit microbial denitrification [22, 23]. Broman et al. [24], on the other hand, found increased denitrification rates in sediment exposed to small amendments of Cd (1 mg Cd kg^−1^ ww sediment) and suggested that this was a result of fluctuating oxygen conditions and cadmium sulfide formation causing reduced metal bioavailability. The presence of toxic metals has been found to change the diversity and reduce the abundance of denitrifying bacteria [25], which might explain some of the different effects on denitrification rates observed in previous studies. Compared to metals, less is known about how persistent organic contaminants may influence denitrification rates. In a 60-day laboratory study, Lindgren et al. [26] observed lower denitrification rates over time, indicating that petroleum hydrocarbons might decrease denitrification rates. However, it is unknown if and how long-term exposures of benthic microbial communities in the field to historical emissions of both metals and persistent organic pollutants (such as PCDD/Fs) may affect the metabolic capacity of benthic microbial communities for nitrogen transformation processes denitrification and DNRA in the sediment.

In this study, we sampled coastal polluted Baltic Sea sediments with the aim of investigating to what extent the nitrogen transformation by sediment microbial communities is affected by historic industrial emissions of toxic metals and dioxins (PCDD/Fs). To address this aim, we analyzed sediment from the contaminated outer harbor of Oskarshamn in south-west Sweden at two stations with different levels of pollution. For reference sediment, we used one of the Swedish marine field reference sites that has little or no industrial pollution, the Uttervik Bay near the Askö Marine Laboratory in the western Baltic Sea. From the collected sediment, we (i) measured denitrification and DNRA rates and (ii) investigated the microbial community composition, diversity, and relative abundance of genes for nitrogen cycling, metal resistance, and degradation of organic pollutants using a metagenomics approach. We hypothesized that the polluted harbor sediment would have (1) lower denitrification and DNRA production rates than reported for unpolluted sites in the Baltic Sea; (2) a low relative abundances of denitrification and DNRA genes, but high genetic capacity for resistance to metals and to degrade organic pollutants; and (3) different bacterial and archaeal taxonomic diversity when compared with sediment communities from less polluted sediments.

## Materials and Methods

### Field Sampling

Polluted sediment was collected on June 27 in 2020, with the research vessel R/V Electra, from the outer harbor of the town Oskarshamn, western Baltic Sea, Sweden. This harbor has a 100 years of history of pollution of metals and persistent organic contaminants due to an adjacent copper plant (no longer active), a Cd-nickel (Ni) battery factory, and shipyards [27]. While the sediment in the inner harbor (not sampled in this study) has been suction-dredged to reduce contamination levels of metals and some of the highest PCDD/PCDFs concentrations so far noted in Sweden [27–31], the outer harbor sediment, which we sampled in this study, has not been remediated [for a map of the harbor area, see [32]. The outer harbor sediments have been reported by environmental consultants to have high concentrations of metals (e.g., Cu, Zn, and Pb with values > 5 times above national reference standard values) and organic pollutants such as dioxins [33]. Sediment was collected from the outer harbor for denitrification and DNRA rate measurements (isotope pairing technique (IPT) incubations), DNA extractions, total organic carbon (TOC) and nitrogen (TN), and metals and PCDD/PCDFs analyses. Samples were collected from two locations ca 130 m apart in the outer harbor: station OSK 1 (16 m water depth; WGS84 coordinates: Lat 57.264483, Long 16.481950; Fig. S1) and station OSK 2 (15.5 m water depth; WGS84 coordinates: Lat 57.265733, Long 16.482300). The sediment was collected using a modified box corer [34] with insert transparent acrylic liners (surface area: 756.25 cm^2^; length and width: 27.5 × 27.5 cm). To collect undisturbed bottom water and sediment, the box cores were subsampled on deck immediately after collection using small transparent acrylic sediment tube cores (cylinders; inner diameter 4.6 cm, length 30 cm). From each station, two box cores were collected. Nine tube cores were subsampled from the first box and 8 tube cores were sampled from the second box. For the station OSK 1, four cores from box 1 and three cores from box 2 were sliced directly on the boat (top 0–1 cm sediment surface) into 50-mL centrifuge tubes (Sarstedt), while for station OSK 2 four cores that were sampled from box 1 were sliced. The sediment samples were stored at − 20 °C and were later used for DNA extraction (OSK 1 *n* = 7, OSK 2 *n* = 4). The remaining sampled sediment cores that had not been sliced (*n* = 10 per station) were closed with rubber stoppers, kept at 8 °C overnight and later used for the ^15^NO_3_^−^ IPT incubation to measure denitrification and DNRA rates. Bottom water (~ 25 L per station) was collected before the sediment collection using 10-L Niskin bottles connected to a Rosette sampler. This water was later used to submerse the sediment cores for the IPT incubation in the laboratory. In parallel, a CTD connected to the Rosette was used to measure in situ temperature, oxygen, and salinity profiles of the water column (SEABird SBE 911 plus). All samples for DNA extraction were transported back to the university on dry ice, and intact sediment cores were transported in a cooling box filled with ice packs. Surficial sediment samples (0–4 cm) for PCDD/Fs and metal analyses (As, Ba, Cd, Co, Cr, Cu, Hg, Ni, Pb, V, Zn) were collected on the same day from additional boxes containing bottom water and sediment (one box was collected from each station using the box corer as described above). The boxes were part of another experiment and were kept aerated until November 18 at 8–16 °C before being subsampled. A total of four randomly selected locations inside each box were subsampled using a syringe (5 mL sediment from each spot), pooled and stored at − 20 °C until further analysis.

An identical sampling approach using the same research vessel, sampling equipment, and type of material was conducted at the Swedish national marine monitoring reference site stations, near the Askö Laboratory station [35, 36], in the Uttervik Bay: station ASK (17 m water depth, WGS84 coordinates: Lat 58.843577, Long 17.544748; Fig. S1), on August 10 during 2020. There is no industry in this area and ASK has no known history of being affected by local metal pollution sources and previous Cd concentration measurements have shown Cd concentrations of ~ 19 µg kg^−1^ dry weight (dw) at the 0–1 cm sediment surface [24], which is well below the national threshold of 0.5 mg kg^−1^ dw set by the Swedish Environmental Protection Agency [37]. Similarly to OSK 1, two box cores were collected and 9 tube cores were subsampled from the first box while 8 tube cores were subsampled from the second box. Four of the cores from box 1 and three cores from box 2 were sliced directly on the boat to be used for DNA extraction (*n* = 7), while the remaining 10 cores were used for IPT incubation. Water column properties were determined and bottom water was sampled as described above. Finally, one additional box was collected, kept aerated, and subsampled for metals and PCDD/Fs as described above.

### Denitrification and DNRA Rate Measurements

Following sampling, the ten collected cores that were not used for slicing were stored, kept in darkness at 10 °C, and submersed in a bath consisting of collected in situ bottom water that was aerated with air stones for 6 days. Externally driven magnetic bars inside the cores were used to continuously mix the water inside the cores to ensure aeration. To increase microbial activity during the IPT incubation, the temperature was changed to 13 °C until the IPT incubation started 5 days later.

The IPT incubation followed the experimental design described by Nielsen [38] and closely followed the protocol described in Broman et al. [24]. In brief, the cores were spiked with ^15^NO_3_ and pre-incubated for 1.5 h to allow ^15^N to homogenize with the in situ ^14^N inside the cores [39]. The cores were then closed using a rubber stopper and incubated for 6 h, followed by collection of water samples from each core for N_2_ analysis (*n* = 9 per station) and dissolved ^15^NH_4_^+^ (*n* = 9 per station). Full details of the methods used for the IPT incubation are available in the supplementary information.

### Sediment Characteristics and Water Analyses

Sediment porosity was measured on a portion of sampled sediment collected in the field (0–1 cm) and was calculated by dividing the water content (sediment dried at 60 °C for 3 days) by the known bulk volume (5 mL). Organic carbon and nitrogen content were measured by acidifying dry sediment (20 mg) with HCl (1 M) over night at 60 °C to remove inorganic carbon and analyzed using a C/N elemental analyzer (Thermo Scientific Flash 2000). Samples for metal analyses were sent to ALS Scandinavia, Sweden and analyzed according to the standards ISO 11464:2006 [40] and SS-EN ISO 17294–2:2016 [41]. In brief, the sediment was dried, sieved (< 2 mm), heated and digested in 7 M HNO_3_, and analyzed by inductively coupled plasma (ICP) (see Table 1 for a list of metals that were measured). Because of the large difference in contaminants between the sites (i.e., much less at ASK), the ASK station required more sediment for PCDD/F analysis. Therefore, in addition to the samples collected with syringes, the ASK box was also subsampled on January 25 in five random locations with acrylic cores (inner diameter: 4.6 cm, length: 30 cm length) and the top 0–3 cm sliced (ca 50 mL sediment per slice) and pooled. Samples for PCDD/Fs were sent to OEKOMETRIC GmbH, Germany and analyzed according to the SS-EN 16,190:2019 standard [42]. In brief, samples were dried, extracted with toluene using pressurized liquid extraction (ASE 180 °C, 140 bar), multicolumn clean-up (multi-silica, ALOX), and analyzed using a gas chromatography high-resolution mass spectrometry (GC-HRMS) (see Table 2 for a list of dioxins and furans measured). A total of 17 congeners were analyzed known to be of toxic relevance and bio-accumulative by organisms in marine sediments [43].

**Table 1:** Field variables measured in situ (bottom water: temperature, salinity, O_2_), subsamples taken from the collected bottom water directly before the IPT experiment (NO_2_^−^ + NO_3_^−^, NH_4_^+^; *n* = 3), surface layer (0–1 cm) sediment variables (porosity, C_org_, N), and measured concentrations of metals in the sediment (0–4 cm surface layer). All heavy metal values are shown as mg kg^−1^ dw

	OSK 1	OSK 2	ASK	NRV*
Temperature °C	16.05	16.05	17.17	
Salinity (ppt)	7.21	7.21	6.61	
O_2_ (mg L^−1^)	6.27	6.27	8.11	
NO_2_^−^ + NO_3_^−^ (µM)	1.5 ± 0.4	1.5 ± 0.4	0.6 ± 0.5	
NH_4_^+^ (µM)	1.1 ± 0.1	1.1 ± 0.1	1.2 ± 1.1	
Porosity (%)	86	90	90	
C_org_ (%)	7.03	9.37	5.00	
N (%)	0.84	1.16	0.68	
C_org_/N ratio	8.37	8.08	7.35	
As	12.1	13.2	5.4	17.0
Ba	54.1	74.7	114.0	–
Cd	3.0	2.5	0.4	0.5
Co	9.1	8.2	14.5	20.4
Cr	26.0	30.8	61.2	48.0
Cu	86.5	66.8	44.8	30.0
Hg	1.4	0.3	< 0.2	0.12
Ni	29.9	34.8	40.7	45.0
Pb	104.0	61.3	34.6	40.0
V	31.8	34.0	63.6	–
Zn	227.0	138.0	175.0	127.5

^*^Heavy metal values considered high with a significant deviation from national reference values (NRV) according to Swedish EPA (2000). Dashes denote that no reference value is available

**Table 2:** Measured concentrations of PCDD/Fs in the sediment (0–4 cm surface layer). All values are shown as ng kg^−1^ dw. Note that there are no national reference values for these compounds in sediments

	PCDD/F	OSK 1	OSK 2	ASK
PCDDs	Total TetraCDD	16	8	11
	Total PentaCDD	26	13	15
	Total HexaCDD	30	21	33
	Total HeptaCDD	41	16	41
	2,3,7,8-TCDD	< 0.4	< 0.4	< 0.4
	1,2,3,7,8-PeCDD	1	0.5	0.9
	1,2,3,4,7,8-HxCDD	1.9	0.7	0.9
	1,2,3,6,7,8-HxCDD	2.2	1.1	2.3
	1,2,3,7,8,9-HxCDD	1.8	0.8	1.6
	1,2,3,4,6,7,8-HpCDD	21	8.3	19
	OCDD	91	32	66
PCDFs	Total TetraCDF	112	60	48
	Total PentaCDF	164	65	59
	Total HexaCDF	203	76	32
	Total HeptaCDF	276	90	26
	2,3,7,8-TCDF	7.8	2.9	4.2
	1,2,3,7,8-PeCDF	11	4.5	2.7
	2,3,4,7,8-PeCDF	8.1	3.3	4.5
	1,2,3,4,7,8-HxCDF	37	12	3.6
	1,2,3,6,7,8-HxCDF	19	6.7	3.8
	1,2,3,7,8,9-HxCDF	3.2	1.1	< 0.4
	2,3,4,6,7,8-HxCDF	11	5.4	3.7
	1,2,3,4,6,7,8-HpCDF	166	52	17
	1,2,3,4,7,8,9-HpCDF	29	9.6	1.9
	OCDF	693	208	23

Samples from the water phase of the cores were analyzed for NH_4_^+^ and NO_2_^−^ + NO_3_^−^ that were measured with standard colorimetric methods indophenol-blue and naphthalene-based azo dye, respectively [44], using a spectrophotometer.

### DNA Extraction and Sequencing

DNA was extracted from 2 g of sediment using the DNeasy PowerMax Soil kit (Qiagen), and the DNA was stored at − 20 °C in 2 mL elution buffer (solution C6). DNA was precipitated and concentrated × 10 with NaCl, EtOH, and Tris as described in the DNeasy PowerMax Soil kit. The final DNA quality and quantity were determined with a NanoDrop 1000 and Qubit 1 (ThermoFisher), respectively. The extracted DNA was prepared with the TruSeq PCR-free kit (Illumina) and sequenced at Science for Life Laboratory at Stockholm on one NovaSeq6000 S4 lane using a 2 × 150 paired-end setup. Sequencing yielded on average 104.9 million reads (min: 80.9, max: 133.8). See Data S1 for a list of sample names and obtained number of sequences per sample. The raw sequence data has been uploaded to the NCBI BioProject PRJNA721298.

### Bioinformatic Analysis

The sequence data was quality trimmed as described previously using SeqPrep and trimmomatic [7]. The functional gene annotation followed the SAMSA2 pipeline [45]. The taxonomic annotation was done on 16S rRNA reads extracted from the metagenomes using the software combo Kraken + Bracken2 [46, 47] as described in Broman et al. [48]. Full details of the bioinformatic analyses are available in the supplementary information.

### Statistics

Shannon’s *H* alpha diversity index was calculated from the 16S rRNA gene data using the software Explicet by subsampling to the lowest sample size (16S rRNA gene data: 30,819 counts) and with bootstrap × 100 (with the mean reported). Beta diversity was tested using the Bray–Curtis dissimilarity index and Sørensen-Dice coefficient and pairwise PERMANOVA tests (9999 permutations) with Bonferroni corrected *P* values using the software past 4.05 [49]. For the beta diversity analyses, the input data was based on either relative abundance (bacteria and archaea, Bray–Curtis) or normalized read counts for the KEGG KO identifiers. Shapiro–Wilk test was used to check if data had a normal distribution using R 3.6.2 [50] with the shapiro.test function (R package: car 3.0–11 [51]) and the homogeneity of variance was analyzed with Levene’s test using the leveneTest function (R package: stats 3.0–4). One-way ANOVA and post hoc Tukey tests were used to test significant differences between the stations using R with the res.aov and TukeyHSD functions. Boxplots with jitter alongside one-way ANOVA and *t*-test were produced with the R package ggpubr 0.4.0. Comparisons of As, Cd, Cu, and Zn metal-related genes between stations were based on listed genes in Yan et al. [52]. The R package Hmisc 4.7.0 [53] was used to conduct Pearson correlations between the normalized read counts for nitrogen cycling genes, and the sum of the metal-related genes, and the sum of dioxin degradation genes. In addition, the edgeR package in R [54] was used to normalize counts (as CPM) and test for statistical differences (false discovery rate, *FDR* < 0.05) between KEGG KOs by using the raw count data as input. All data are reported as mean ± standard error.

## Results

### Field Conditions, Metal and PCDD/F Concentrations in the Sediments

The sampled polluted area in Oskarshamn (stations OSK 1 and OSK 2) and the national reference station (ASK) both had oxic bottom waters, salinity 6.27–7.21, and a temperature of 16.05–17.17 °C (Table 1). Dissolved NO_2_^−^ + NO_3_^−^ was higher in the bottom water at Oskarshamn compared to the reference station ASK (*n* = 3; one-way ANOVA, df = 5, *F* = 223.9, *P* < 0.001), while dissolved NH_4_^+^ concentrations in the bottom water were similar among stations (*n* = 3; one-way ANOVA, df = 5, *F* = 0.204, *P* = 0.675). The sediment porosity (Φ) was similar for each station, while sediment organic carbon (C_org_) and nitrogen content (N) were different among all stations (*n* = 1 per station; Table 1).

Harbor sediments at OSK 1 and 2 were found to contain twice as high concentrations of As, Cu, Hg, Pb, and Zn, respectively, and seven times higher concentrations of Cd when compared with the reference site ASK (based on the average for OSK 1 and OSK 2, Zn was only higher at OSK 1; Table 1). The highest concentrations of Cd (3.0), Cu (86.5), Hg (1.4), Pb (10.40), and Zn (227.0) were found at OSK 1 (values shown as mg kg^−1^ dw). Compared with the two OSK stations, the sediment at ASK contained higher concentrations of Ba, Co, Cr, Ni, and V (Table 1). However, most of these metals at ASK were below what is classified as a high significant deviation from the national threshold by the Swedish Environmental Protection Agency, except for Zn and Cu at all stations. Notably, Cd, Hg, and Pb were categorized as “high” at the OSK stations; however, Cr was categorized as “high” at the ASK station (Table 1). For dioxin concentrations, taken together, there were no large differences between the two OSK stations as compared with ASK (on average 0.6–1.3 times difference per compound); however, concentrations were higher at OSK 1 than at OSK 2 (Table 2). In contrast, furan concentrations were on average 7 and 3 times higher at OSK 1 and OSK 2, respectively, compared with ASK (Table 2). The highest concentrations of furans were octachlorodibenzofuran (OCDF) and total HeptaCDF with OCDF at OSK 1 (693.0), OSK 2 (208.0), ASK (23.0), and total HeptaCDF at OSK 1 (276.0), OSK 2 (90.0), and ASK (26.0), respectively (all values shown as ng kg^−1^ dw).

### Denitrification and DNRA Rates

**Fig. 1:**
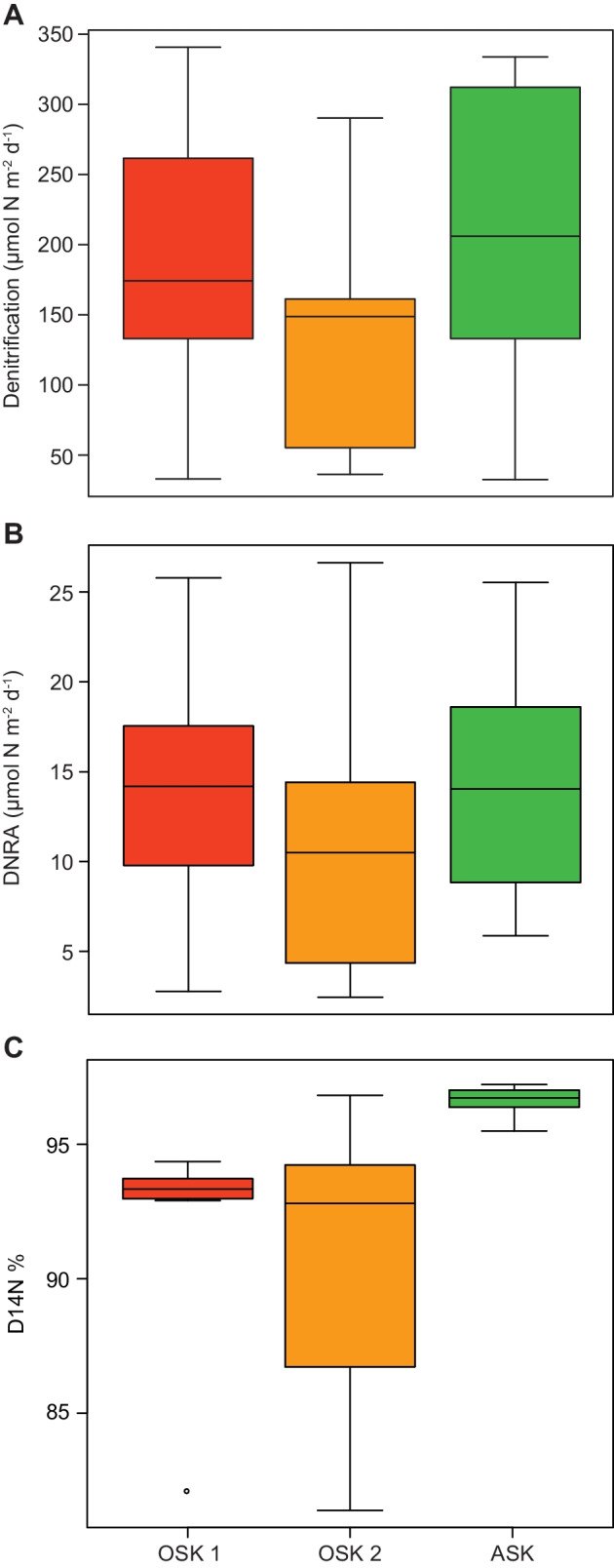
Boxplots showing N_2_ production rates from ^15^NO_3_.^−^ labeled incubations conducted on sediment sampled from the polluted harbor of Oskarshamn (stations OSK 1 and OSK 2) and a reference station Askö (station ASK). The subpanels show **A** denitrification rates; **B** DNRA rates; and **C** denitrification derived from nitrification (D14N). The middle line inside each box denotes the median, the top and bottom of each box represent the 25th and 75th quartile, while the whiskers represents the maximum and minimum values. Number of replicates *n* = 9 per station except for **A** OSK 1 denitrification *n* = 8 and **C** OSK 1 D14N *n* = 8

The ^15^NO_3_^−^ isotope labeling incubations showed that there was no significant difference in denitrification rates among the stations (OSK 1 189.1 ± 30.4, OSK 2 131.9 ± 23.4, and ASK 210.6 ± 33.0 µmol N m^−2^ d^−1^; one-way ANOVA, df = 25, *F* = 1.59, *P* = 0.23, *n* = 9 per station except OSK 1 *n* = 8; Fig. 1A). This represents 92.3, 92.6, and 93.7% denitrification of the total nitrate reduction, respectively (i.e., % denitrification = denitrification / (denitrification + DNRA)). Similarly, no significant difference in DNRA rates was found among the stations (OSK 1 15.0 ± 2.2, OSK 2 11.0 ± 2.4, and ASK 14.2 ± 2.0 µmol N m^−2^ d^−1^; one-way ANOVA, df = 26, *F* = 0.75, *P* = 0.48; *n* = 9 for each station; Fig. 1B). This represents 7.7, 7.4, and 6.3% DNRA of the total nitrate reduction, respectively (i.e., % DNRA = DNRA / (denitrification + DNRA)). See Data S2 for a full list of all data for each replicate. Even though no significant differences were found for denitrification and DNRA rates, the calculated denitrification derived from nitrification (D14N) was higher at the reference station ASK (96.9 ± 0.3%) compared with the polluted harbor stations OSK 1 (92.1 ± 0.7%) and OSK 2 (90.9 ± 0.8%) (one-way ANOVA tests, df = 16 and 17, *F* = 11.9 and 12.6, *P* < 0.01 for both OSK 1 and OSK 2; Fig. 1C).

### Nitrogen Cycling Pathways and Genetic Signatures

Metagenome data analysis resulted, on average, in 19.8 million reads (range: 14.7–25.6) per sample that could be classified to KEGG KO identifiers (assigned reads in MEGAN). After normalization (subsampling to lowest sample size), on average 173,000 reads could be assigned to KEGG KO identifiers belonging to the nitrogen metabolism pathway. A full list of KEGG nitrogen metabolism classifications with normalized read counts is available in Data S3. Full pathways for DNRA, assimilatory nitrate reduction, denitrification, N_2_ fixation, nitrification, and anammox were identified at all three stations (Fig. S2). Analysis of the Bray–Curtis dissimilarity index of all KEGG KO identifiers in the nitrogen metabolism map showed that the stations were different from each other (PERMANOVA, df = 17, pseudo-*F* = 22.17, *P* < 0.001; with all stations being different from each other when tested with pairwise comparison, Bonferroni corrected *P* values < 0.05).

**Fig. 2:**
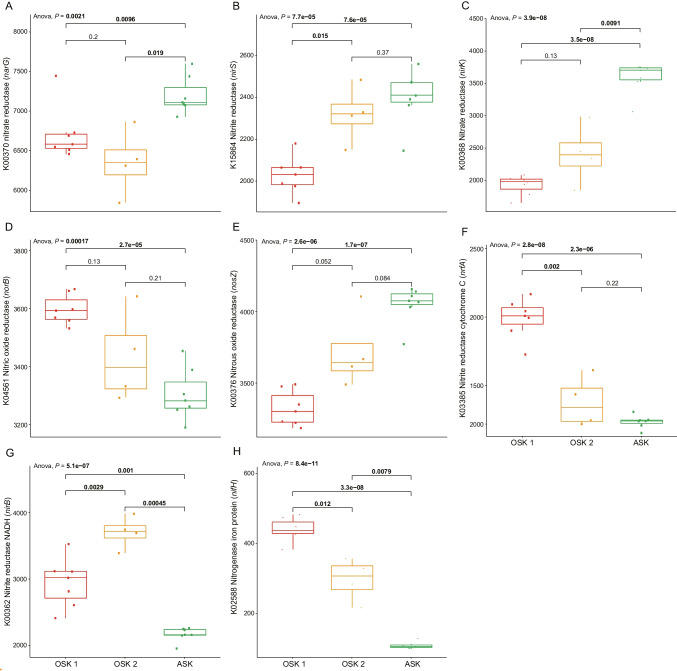
Boxplots with jitter showing selected nitrogen cycling KEGG KO identifiers and their associated gene names, each sample is indicated as a dot (i.e., jitter). **A** shows nitrate reduction (*narG* gene), two genes for nitrite reduction **B** *nirS* and **C** *nirK*, **D** nitric oxide reduction (*norB*), **E** nitrous oxide reduction (*nosZ*), two genes for DNRA **F** *nrfA* and **G** *nirB*, and **H** nitrogen fixation (*nifH*). The data is based on normalized read counts, and the one-way ANOVA results show the results for the whole model (including all three stations), while the *P* value between stations is based on pairwise *t*-tests. *P* values < 0.05 have been marked with bold text. The middle line inside each box denotes the median, the top and bottom of each box represent the 25th and 75th quartile, while the whiskers represents the maximum and minimum values. Outliers are shown outside the whiskers if the distance is longer than 1.5 × box height. Number of replicates: ASK *n* = 7, OSK 1 *n* = 7, OSK 2 *n* = 4

The majority of nitrogen cycling genes that could be classified to taxa were attributed to different Proteobacteria classes (Alpha, Beta, Delta, Gamma), Bacteroidetes, and archaeal Thaumarchaeota at all stations (Data S4). Looking closer at abundances of genes for reductive N-cycling processes, differences were observed both for denitrification and DNRA. The statistical tests, edgeR, and one-way ANOVA with *t*-tests showed similar results and the one-way ANOVA tests are reported below (see methods for more details and Data S5 for edgeR results). Polluted harbor sediments OSK 1 and OSK 2 showed a lower relative abundance of *narG*, *nirS*, and *nirK* genes, as well as *nosZ* for OSK 1, when compared to the reference station ASK (Student’s *t*-test, *P* < 0.05; Fig. 2A–E). Analyses of differences in the denitrification pathways within the polluted stations showed that there was no difference in nitrate reduction genes between OSK 1 and OSK 2 (for example, the *narG* gene; Fig. 2A); nitrite reduction was significantly different only for *nirS* gene abundance but not *nirK* (with more *nirS* genes in OSK 2 compared to OSK 1; one-way ANOVA Student’s pairwise *t*-test, *P* < 0.05, Fig. 2B–C), while nitric oxide and nitrous oxide reduction (*norB* and *nosZ*, respectively) showed no difference between the two OSK sites (Fig. 2D–E). However, the *nirS* and *nirK* community composition was similar between OSK 1 and OSK 2 with most of the classified genes attributed to Gammaproteobacteria, Thaumarchaeota, and Chloroflexi (Fig. S3 and Data S4). In addition, more *nirK* genes were attributed to the archaeal phylum Thaumarchaeota at ASK when compared with both OSK 1 and OSK 2 (one-way ANOVA tests, *F* = 177 and 91, respectively, *P* < 0.01; Data S4). General differences in taxonomic profile attributed to these genes included a higher relative abundance of Nitrospirae carrying the *narG* gene at ASK, whereas at OSK 1 the *narG* gene was predominantly attributed to Deltaproteobacteria (Data S4). Most of the *nosZ* genes could not be classified (ca 60%) but the majority of the sequences that could be classified belonged to Bacteroidetes, with no difference among stations (Data S4).

For sequence reads that could be matched to DNRA, there were more identified genes coding for nitrate reductase cytochrome C (*nrfA*) at OSK 1 compared with both ASK and OSK 2 (Student’s *t*-test, *P* < 0.05: Fig. 2F), while reads for nitrite reductase NADH (*nirB*), another enzyme in DNRA, were overrepresented at OSK 2 compared with ASK and OSK 1 (Student’s *t*-test, *P* < 0.05: Fig. 2G). Within taxa attributed to *nrfA*, the analyses showed that Bacteroidetes had a higher relative abundance at OSK 1 when compared with ASK and OSK 2 (one-way ANOVA tests, *F* = 248 and 11, respectively, *P* < 0.01; Fig. S3 and Data S4). Furthermore, taxa attributed to *nirB* were found to be dominated by Gammaproteobacteria at OSK 1 and OSK 2 but could not be further classified to family level (Data S4). Finally, we also observed a difference between stations in the nitrogen fixation gene *nifH* that was highest at OSK 1 (Fig. 2H), ammonia oxidation genes *amoAB* that were highest at ASK, and anammox genes *hzs* and *hdh* that had low counts (< 30) at all stations (Fig. S4). See supplementary information for full details of these additional results.

### Metal and PCDD/F Resistance and Genetic Signatures

**Fig. 3:**
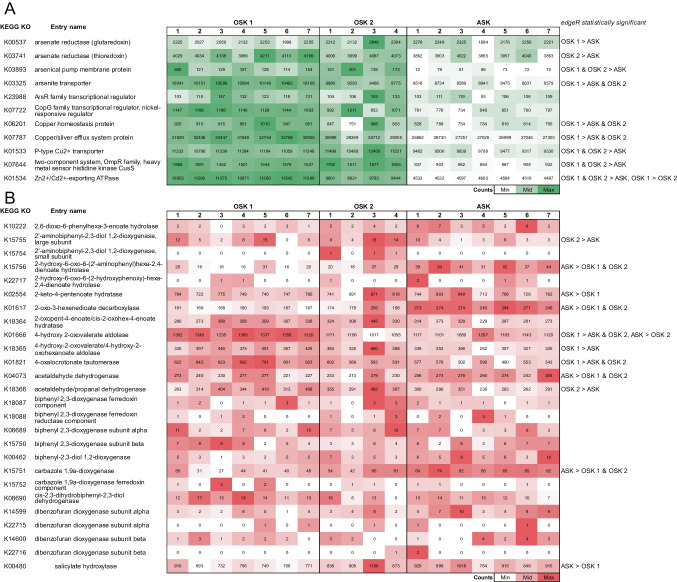
Heatmap of **A** KEGG KOs attributed to metal-related genes for As, Cd, Cu, and Zn for each station and replicate sample (denoted with a number on the top x-axis) and **B** KEGG KOs included in the dioxin degradation KEGG pathway, respectively. The list of selected genes for the metals is based on Yan et al. [52]. The data shown is based on normalized read counts. The edgeR results show which KEGG KOs were significantly higher between stations (*FDR* < 0.05). Note that the color gradients are based on the lowest and highest values per row

Both polluted harbor stations OSK 1 and OSK 2 were found to have higher numbers of reads attributed to several metal-related KEGG KOs compared with the reference station ASK, e.g., genes encoding for arsenate reductase (glutaredoxin/thioredoxin), P-type Cu^2+^ transporter, two-component system OmpR family heavy metal sensor histidine kinase CusS, and Zn^2+^/Cd^2+^-exporting ATPase (edgeR, *FDR* < 0.5; Fig. 3A). Furthermore, the relative abundance of reads attributed to some of these genes was higher at OSK 1 compared with OSK 2, including the genes coding for copper homeostasis protein, copper/silver efflux system protein, and Zn^2+^/Cd^2+^-exporting ATPase, respectively (Fig. 3A). Taxa at OSK 1 and OSK 2 that could be affiliated to the metal-related genes were most closely related to members of the order Desulfuromonadales (that was also attributed to N_2_-fixation, *nifH*) and were classified to, e.g., P-type Cu^2+^ transporter, two-component system OmpR family heavy metal sensor histidine kinase CusS, Zn^2+^/Cd^2+^-exporting ATPase, and copper/silver efflux system protein (Data S4). Several phyla and classes that were attributed to denitrification and DNRA genes were also affiliated with the metal-related genes included, e.g., Bacteroidetes, Chloroflexi, Alpha-, Delta-, and Gammaproteobacteria (Data S4). Examples of taxa that both denitrification (*nirS* or *nirK*) or DNRA (*nrfA* or *nirB*) and metal-related genes could be affiliated with included *Pseudomonas*, Thiotrichaceae, Gemmatimonadaceae, Rhodobacteraceae, Desulfobacterales, and Cytophagales (Data S4).

The dioxin degradation pathway included dibenzofuran dioxygenase enzymes involved in both PCDD and PCDF degradation with genes *dxnA1*, *dxnA2*, *dbfA1*, and *dbfA2* (KEGG KOs: K22715, K22716, K14599, K14600, respectively). None of these KEGG KOs was, however, statistically significantly different among stations (Fig. 3B). Nor was there any clear pattern that showed that the harbor stations had a higher gene abundance for later steps in these pathways (Fig. 3B). Overall, reads attributed to PCDD and PCDF degradation were significantly fewer than those attributed to metal-related genes (~ 5000 reads per sample compared to ~ 65,000 attributed to the toxic metals). Taxa attributed to dibenzofuran dioxygenase enzymes included mainly Actinobacteria and Gammaproteobacteria with no further classification to class level (Data S4).

Pearson correlations based on the normalized read counts for the metal resistance, dioxin degradation, and the nitrogen cycling genes showed that *narG*, *nirS*, *nirK*, and *nosZ* were negatively correlated with the sum of all metal-related genes (*r* = − 0.50, − 0.70, − 0.91, − 0.83, all *P* < 0.05, respectively). In contrast, the genes *norB*, *nrfA*, and *nirB* were positively correlated with the metal-related genes (*r* = 0.85, 0.84, 0.67, all *P* < 0.05, respectively). The sum of the dioxin degradation genes did not correlate significantly with any of the nitrogen cycling genes.

### Microbial Diversity and Community Composition

Considering the difference in pollution between the harbor stations and the national reference station, we also investigated whether there were differences in taxonomic diversity among the bacterial and archaeal communities. Shannon’s *H* diversity index was significantly lower at the reference ASK station (6.16 ± 0.05 Shannon’s *H*, *n* = 7) when compared with both OSK 1 (6.82 ± 0.04, *n* = 7) and OSK 2 (6.87 ± 0.04, *n* = 4) (tested on the 16S rRNA gene data; one-way ANOVA, df = 17, *F* = 82.4, *P* < 0.001; and with post hoc TukeyHSD multiple comparison test: *P* < 0.001 for both harbor stations when compared with ASK). There was, however, no difference in Shannon’s *H* diversity index between the two harbor stations OSK 1 and OSK 2 (one-way ANOVA with post hoc Tukey test, *P* = 0.81). The Bray–Curtis dissimilarity index was tested between all stations and showed that there was a significant difference in community composition (PERMANOVA, df = 17, pseudo-*F* = 20.98, *P* < 0.001). Pairwise comparison showed that the ASK station was different from both OSK 1 and OSK 2 (Bonferroni corrected *P* values < 0.05), and that OSK 1 and OSK 2 were different (Bonferroni corrected *P* values < 0.05).

**Fig. 4:**
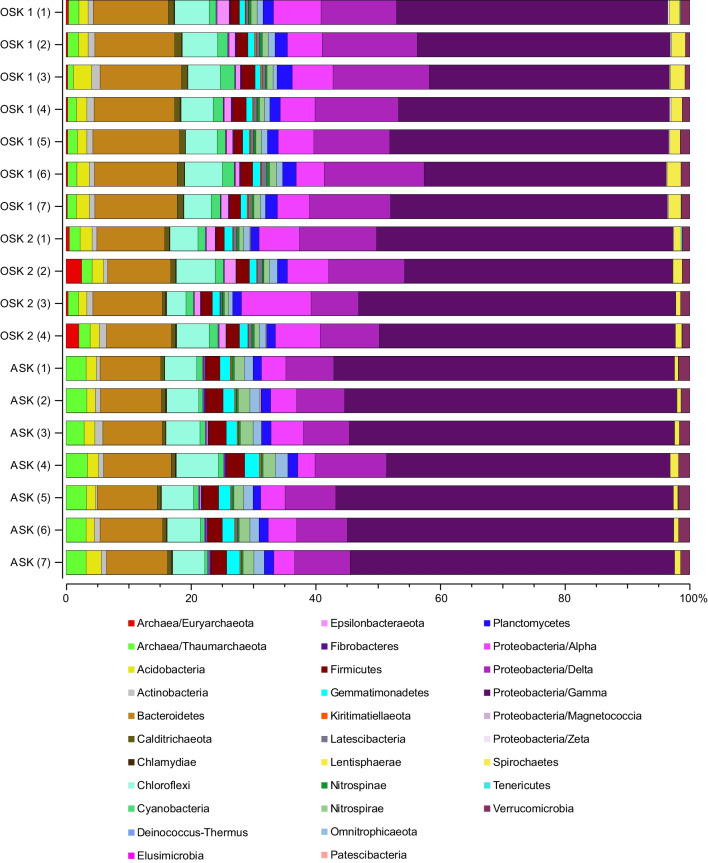
Stacked bars showing the bacterial and archaeal community composition as relative abundance (%) detected in the collected sediment samples (top 1 cm sediment slice). The data is based on metagenome extracted 16S rRNA gene sequences classified against the SILVA database (using the Kraken2 + Bracken2 software combo). OSK 1 = Oskarshamn station 1, OSK 2 = Oskarshamn station 2, ASK = Askö station. The numbers in parenthesis denote the replicate number (individual sediment cores)

**Fig. 5:**
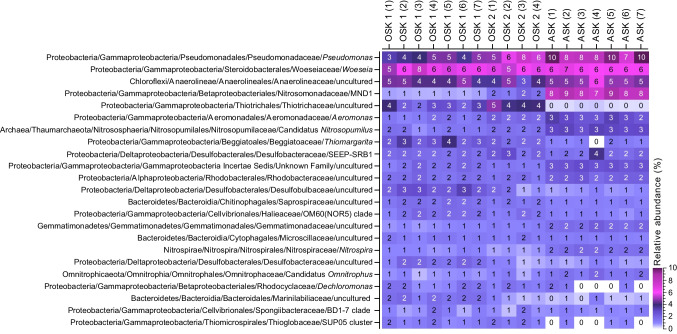
Heatmap showing the dominant bacterial and archaeal taxa in the sediment based on the metagenome 16S rRNA gene data. The heatmap shows data based on lowest classified taxonomic level (i.e., genera). The data was delimited to genera > 1% (average of all samples). OSK 1 = Oskarshamn station 1, OSK 2 = Oskarshamn station 2, ASK = Askö station. The numbers in parenthesis denote the replicate number (individual sediment cores). The labels inside the heatmap denote the relative abundances (%), with dark cells having white labels to enhance reading clarity

Overall, the bacterial and archaeal communities were dominated by Proteobacteria (classes Gamma, Delta, and Alpha in order of highest relative abundance) at all three stations. Additional top dominant phyla included Bacteroidetes, Chloroflexi, Firmicutes, and Thaumarchaeota (Fig. 4). Closer analyses of some examples of the dominant genera that were different among stations showed that *Pseudomonas* had the highest relative abundance at ASK (on average 8.7%), followed by OSK 2 (6.0%) and OSK 1 (4.1%) (one-way ANOVA, df = 17, *F* = 37.96, *P* < 0.05; with post hoc TukeyHSD test, *P* < 0.05 for all three stations; Fig. 5). An uncultured genus belonging to the family Anaerolineaceae had a higher relative abundance at station ASK (5.0%) when compared with OSK 2 (3.8%) (*F* = 5.089, *P* < 0.05, TukeyHSD, *P* < 0.05) but there was no major difference between OSK 1 (4.4%) and OSK 2. The MND1 genus belonging to the family Nitrosomonadaceae was significantly different among all stations, with the lowest relative abundance in the polluted sediment at OSK 1 (0.7%) and OSK 2 (1.7%) compared with the reference station ASK (8.1%) (*F* = 811.3, *P* < 0.001; TukeyHSD, *P* < 0.05; Fig. 5). An uncultured genus belonging to the family Thiotrichaceae was also significantly different among all stations, most abundant at OSK 2 (4.2%) followed by OSK 1 (2.7%), and lowest at ASK (0.3%) (*F* = 73.61, *P* < 0.001; TukeyHSD, *P* < 0.05; Fig. 5).

Moreover, the class Dehalococcoidia that consists of known bacterial members capable of carrying out dechlorination had a higher relative abundance at the OSK 1 and OSK 2 stations when compared with ASK (one-way ANOVA tests, df = 13 and 10, *F* = 138.5 and 50.0, *P* < 0.01 for both, respectively). The relative abundance of Dehalococcoidia was, however, low at all stations with ASK 0.01 ± 0.02%, OSK 1 0.27 ± 0.05%, and OSK 2 0.36 ± 0.15%. A full list of all taxonomy classifications with read counts can be found in Data S6.

## Discussion

This study shows that the benthic microbial community from the historically polluted sediment in Oskarshamn’s outer harbor has adapted to the metal pollution. These results reflect the harbor’s history of pollution over the last century [55], with historic emissions of contaminants derived from various industrial processes, e.g., a copper plant operated between 1918 and 1969, and a battery factory (since 1917, still in operation) [27]. While the inner harbor has recently been remediated through suction-dredging to decrease the level of pollutants [29–31], the outer harbor remains unremedied and, as this study shows, is still polluted with high levels of metals and PCDD/Fs. Concentrations of Cd, Cu, Hg, and Pb in the harbor sediments were significantly higher compared with the national reference station near Askö (ASK), and significantly above the national thresholds [37]. Furans (PCDF) concentrations were 3–7 times higher at the harbor stations (OSK) compared with ASK. Calculated PCDD/F toxic equivalencies (ng kg^−1^ TEQ dw) showed that OSK 1 (15 ng kg^−1^) was three times higher than at both OSK 2 (5 ng kg^−1^) and ASK (5 ng kg^−1^) [TEF values to calculate TEQ taken from 56]. Given the large differences in pollution between polluted OSK stations and the national reference station, and considering that mixed metal pollution, as well as petroleum exposure experiments, have been shown to decrease the rate of denitrification [22, 23], we expected lower denitrification and DNRA rates in the polluted harbor sediments. However, we detected no statistically significant differences in denitrification or DNRA rates among the stations.

Observed denitrification and DNRA rates (Fig. 1) were similar to those previously shown at unpolluted sites in the southern and central Baltic Sea [57, 58], which indicates that the polluted stations had denitrification and DNRA rates within the range generally found in the Baltic Sea. In more detail, for the two OSK stations, the average denitrification rates calculated per hour (5.5–7.9 µmol N m^−2^ h^−1^) and DNRA rates (0.5–0.6 µmol N m^−2^ h^−1^) are at similar levels to what has previously been reported for the Himmerfjärden area nearby the reference station Askö [58]. These values are also comparable to denitrification rates measured in the central Baltic Sea and in coastal sediments in the more saline southern Baltic Sea [57]. There was a time difference (of ca 6 weeks between June 27 and August 10), due to logistical reasons, between sampling the OSK and ASK stations; however, our results do not indicate a significant inhibition effect on denitrification and DNRA at the polluted Oskarshamn stations. Moreover, previous research has shown that denitrification rates at Askö station were significantly lower in June than in August [58]; thus, denitrification and DNRA rates would have been expected to be ~ 3 times lower at Askö if the station had been sampled 6 weeks earlier (i.e., in June), further supporting that inhibition was only minor at the OSK stations. In this study, we investigated two stations within one polluted harbor in the Baltic Sea, and potentially other polluted sites with different environmental conditions might yield different results when compared to the reference station. Nevertheless, the high concentrations of contaminations at our polluted sites indicate that our results are likely relevant for other polluted coastal areas. Possibly anaerobic oxidation of ammonium (anammox) rates, which were not analyzed in this study, could contribute to the total nitrate reduction but since several previous studies have shown that anammox activity is typically of minor importance in shallow estuarine and coastal sediments [59], as shown at, for example, Askö [58], the process is not expected to be significant at the Oskarshamn stations. Indeed, the metagenome data indicated a very low capacity for anammox in the studied sediments, with < 30 read counts at ASK and < 15 counts at respective OSK station. Anammox rate measurements to estimate its contribution to total nitrate reduction would be relevant; however, in studies of polluted sediments at deeper water depths, this process has been shown to be of less importance below 100 m water depth [59]. To summarize, our findings suggest that the long-term metal and organochloride pesticide pollution did not have any significant inhibitory effect on reductive N cycling processes at the time of sampling.

The genetic adaptation of the microbial communities at OSK 1 and 2 to the high concentrations of pollutants was revealed by the metagenome results. This was evident by the gene data with an overrepresentation of metal resistance genetic prerequisites at OSK 1 and 2, and the presence of taxa that could be matched to both denitrification/DNRA genes and the metal-related genes, such as Thiotrichaceae and *Pseudomonas*. The family Thiotrichaceae, which was significantly more abundant in the polluted sediments, has previously been described to include members with genetic prerequisites for both metal tolerance and DNRA production [9, 60–62]. The genus *Pseudomonas*, which was present at all stations, is known to include species with both metal tolerance and denitrification capacity [63–65]. However, with the data obtained in this study, it was not possible to differentiate among species of this genus. Results of metal resistance capacity complement previous findings, which showed that Baltic Sea deep water sediments were overrepresented in Cd-Zn-Hg resistance pathways in comparison with several other deep waters and sediments globally [66, 67], and suggest a broader genetic adaptation to metal pollution in the Baltic Sea. Even though we detected differences in denitrification gene abundances (such as *nirK* and *nirS*) among stations, these genes have previously been described to also be present in, e.g., anaerobic ammonium-oxidizing bacteria and methane-oxidizing bacteria [68], which we identified at all stations, possibly partly explaining less correlation with denitrification rates. Here we used the KEGG KO annotations from software combo DIAMOND + MEGAN [69]. We have previously used these tools in conjunction with metagenome assemblies, RNA-seq, and measured environmental variables to validate our findings [70–72]. Differences observed in N-cycling genes in our dataset (e.g., *norB* being high at OSK while *nirS* being high at ASK) are therefore potentially a result of the environmental conditions at each station selecting for specific microbes carrying these genes. Our metagenomics results furthermore confirm that the abundance of genes encoding proteins of enzymes involved in nitrate reduction does not directly relate to the biogeochemical rates, as previously shown for several biogeochemical processes [73].

In addition to microbial community traits, environmental conditions at sampled sites may explain denitrification rates. For example, differences in organic matter content, grain size, and redox potential are more likely than metals to influence denitrification rates [74, 75]. The sampled stations were all oxic at the sampled depth, but presented natural differences in, e.g., temperature, salinity, and marked differences in quantity/quality of organic matter (see C_org_ and N data). Especially this latter factor may explain a part of the variation in our data similarly to what has been previously observed in Baltic Sea sediments [76]. Since there was more C_org_ and N (as well as a higher C_org_/N ratio) at the polluted sites than at the reference site, we argue that the stimulatory effect of more C and N on denitrification rates might have been negatively offset by the high levels of contaminants. Even though NO_2_^−^ + NO_3_^−^ in the bottom water were low at all stations, it was slightly higher at the polluted sites (on average 1.5 µM) compared with the reference site (0.6 µM), which might explain small differences in nitrogen cycling processes between the sites. While O_2_ and NH_4_^+^ concentrations at the bottom water revealed oxic conditions, O_2_, H_2_S, and NH_4_^+^ concentrations in the sediment surface were not determined and differences in the sediment redox profile among stations might also have influenced denitrification and DNRA rates. However, we have a good grasp of how much these sediments consume in terms of O_2_ consumption over time since extensive microprofiling in all these sediment has been conducted in our previous works [58, 76–79]. When pre-incubating the sediments for 1.5 h followed by 6 h at 13 °C, these sediments are estimated to consume on average 27% of the initial oxygen available in the cores, meaning that no cores went hypoxic. Also, due to the very low oxygen penetration depth of only a mm [see, for example, 78], N_2_ production rates were likely linear over time. Furthermore, we observed higher denitrification derived from nitrification (i.e., D14N) at ASK compared with OSK 1 and OSK 2, which was also in accordance with a higher relative abundance of *amoABC* gene counts, and Nitrosomonadaceae bacteria at ASK. Even though the cores were “flooded” with N during the IPT incubation (an increase from ~ 0.6 µM to ~ 20 µM NO_3_^−^), this could indicate that nitrification limitation is regulating denitrification rates at ASK even under ambient NO_3_^−^ concentrations [80].

Even though there was an obvious difference in metal resistance genes between the polluted stations and the national reference station, the metagenome data showed that the capacity to degrade PCDD/Fs in the sediment was limited at all three studied stations. The latter result was less expected as there are known specific bacterial populations, such as *Pseudomonas* species, that have been shown to tolerate PCDD addition [81], and some bacteria, such as *Dehalococcoides*, degrade PCDD/Fs (so-called dehalogenation) using hydrogen as an electron donor [82–84]. That there was no observed difference in denitrification rates among the studied sites suggests that the PCDD/Fs not noteworthy influenced the nitrate-reducing populations. Explanations for a limited capacity to degrade PCDD/Fs in our dataset include, e.g., that (1) these substances have a low solubility and therefore sorb to particles [85] resulting in less available substances for the microbial community and (2) bacterial transformation of PCDD/F depends on H_2_ as an electron donor [82, 83], which at the high concentration of sulfate and abundance of sulfate-reducing bacteria (that consume H_2_) possibly made degradation of PCDD/Fs unfavorable [83]. This was also indicated by a low relative abundance attributed to *Dehalococcoides* (< 0.1% per station, Data S6). Potentially, results would be different in freshwater systems where H_2_ might be more readily available at the sediment surface. Our results therefore indicate that microbial transformation of PCDD/Fs was likely not a major metabolic process at any of the studied coastal sites.

## Conclusions

Our findings indicate that benthic denitrification and DNRA do not seem to be affected by historic contaminant emissions of metals and dioxins in Baltic Sea sediment collected during the summer. We found that the polluted sediment at the outer harbor of Oskarshamn holds a N-cycling microbial community that is adapted to pollution. This was indicated by an overrepresentation of metal resistance genes, some of which could be matched to putative denitrifiers, which might partly explain the denitrification rate results. However, the same pattern was not observed for PCDD/Fs degradation genes, which might be due to the fact that the dioxins are adsorbed to sediment particles and have a relatively low bioavailability, or due to sulfate-reducing bacteria competing for H_2_ as an electron donor (that is also used in bacterial transformation of PCDD/Fs). Even though the polluted sediment revealed less sequences attributed to denitrification genes, such as *nirK*, *nirS*, and *nosZ*, this was not reflected in the N-cycling rates when compared to a national reference station and other sites in the Baltic Sea. Our results of denitrification/DNRA rates and metal genes indicate an important adaptation of nitrate-reducing bacteria to metals resulting in a more metal-tolerant microbial community with persistent nitrate reduction in the polluted harbor sediment. Consequently, the microbial N-cycling might be more impacted by the access to organic carbon and nutrients than by pollution of metals and dioxins.

### Supplementary Information

Below is the link to the electronic supplementary material.

Supplementary file1 (XLSX 12 KB)

Supplementary file2 (XLSX 12 KB)

Supplementary file3 (XLSX 8966 KB)

Supplementary file4 (XLSX 1497 KB)

Supplementary file5 (XLSX 1129 KB)

Supplementary file6 (XLSX 55 KB)

Supplementary file7 (DOCX 2.55 MB)

## Data Availability

All data supporting this study is available in the published article or supplementary information. The raw sequencing data has been uploaded to the NCBI BioProject PRJNA721298.

## References

[1] Galloway JN, Dentener FJ, Capone DG, Boyer EW, Howarth RW, Seitzinger SP, Asner GP, Cleveland CC, Green PA, Holland EA, Karl DM, Michaels AF, Porter JH, Townsend AR, Vöosmarty CJ (2004). Nitrogen cycles: past, present, and future. Biogeochemistry.

[2] Lotze HK, Lenihan HS, Bourque BJ, Bradbury RH, Cooke RG, Kay MC, Kidwell SM, Kirby MX, Peterson CH, Jackson JBC (2006). Depletion, degradation, and recovery potential of estuaries and coastal seas. Science.

[3] Carstensen J, Andersen JH, Gustafsson BG, Conley DJ (2014). Deoxygenation of the Baltic Sea during the last century. Proc Natl Acad Sci USA.

[4] Cornwell JC, Kemp WM, Kana TM (1999). Denitrification in coastal ecosystems: methods, environmental controls, and ecosystem level controls, a review. Aquat Ecol.

[5] Payne WJ (1973). Reduction of nitrogenous oxides by microorganisms. Bacteriol Rev.

[6] Hietanen S, Jäntti H, Buizert C, Jürgens K, Labrenz M, Voss M, Kuparinen J (2012). Hypoxia and nitrogen processing in the Baltic Sea water column. Limnol Oceanogr.

[7] Broman E, Zilius M, Samuiloviene A, Vybernaite-Lubiene I, Politi T, Klawonn I, Voss M, Nascimento FJA, Bonaglia S (2021). Active DNRA and denitrification in oxic hypereutrophic waters. Water Research.

[8] Mohan SB, Schmid M, Jetten M, Cole J (2004). Detection and widespread distribution of the nrfA gene encoding nitrite reduction to ammonia, a short circuit in the biological nitrogen cycle that competes with denitrification. FEMS Microbiol Ecol.

[9] Pandey CB, Kumar U, Kaviraj M, Minick KJ, Mishra AK, Singh JS (2020). DNRA: a short-circuit in biological N-cycling to conserve nitrogen in terrestrial ecosystems. Science of The Total Environment.

[10] Burdige DJ (2006) Geochemistry of marine sediments. PRINCETON University Press

[11] Griffiths JR, Kadin M, Nascimento FJA, Tamelander T, Törnroos A, Bonaglia S, Bonsdorff E, Brüchert V, Gårdmark A, Järnström M, Kotta J, Lindegren M, Nordström MC, Norkko A, Olsson J, Weigel B, Žydelis R, Blenckner T, Niiranen S, Winder M (2017). The importance of benthic–pelagic coupling for marine ecosystem functioning in a changing world. Glob Change Biol.

[12] Breitburg D, Levin LA, Oschlies A, Grégoire M, Chavez FP, Conley DJ, Garçon V, Gilbert D, Gutiérrez D, Isensee K, Jacinto GS, Limburg KE, Montes I, Naqvi SWA, Pitcher GC, Rabalais NN, Roman MR, Rose KA, Seibel BA, Telszewski M, Yasuhara M, Zhang J (2018). Declining oxygen in the global ocean and coastal waters. Science.

[13] Howarth RW, Marino R (2006). Nitrogen as the limiting nutrient for eutrophication in coastal marine ecosystems: evolving views over three decades. Limnol Oceanogr.

[14] Qian Y, Zhang W, Yu L, Feng H (2015). Metal pollution in coastal sediments. Current Pollution Reports.

[15] Czuczwa JM, Hites RA (1984). Environmental fate of combustion-generated polychlorinated dioxins and furans. Environ Sci Technol.

[16] Ryu J, Khim JS, Kang S-G, Kang D, Lee C-h, Koh C-h (2011). The impact of heavy metal pollution gradients in sediments on benthic macrofauna at population and community levels. Environ Pollut.

[17] Kang T, Oh JH, Hong J-S, Kim D (2018) Response of intertidal meiofaunal communities to heavy metal contamination in laboratory microcosm experiments. J Coast Res 85:361–365

[18] Somerfield P, Gee J, Warwick R (1994). Soft sediment meiofaunal community structure in relation to a long-term heavy metal gradient in the Fal estuary system. Mar Ecol Prog Ser.

[19] Iburg S, Nybom I, Bonaglia S, Karlson AM, Sobek A, Nascimento FJ (2020) Organic contaminant mixture significantly changes microbenthic community structure and increases the expression of PAH degradation genes. Front Environ Sci 8:128

[20] Nies DH (1999). Microbial heavy-metal resistance. Appl Microbiol Biotechnol.

[21] Ma J, Ullah S, Niu A, Liao Z, Qin Q, Xu S, Lin C (2021). Heavy metal pollution increases CH_4_ and decreases CO_2_ emissions due to soil microbial changes in a mangrove wetland: microcosm experiment and field examination. Chemosphere.

[22] Sakadevan K, Zheng H, Bavor HJ (1999). Impact of heavy metals on denitrification in surface wetland sediments receiving wastewater. Water Sci Technol.

[23] Liu Y, Liu Y, Zhou H, Li L, Zheng J, Zhang X, Zheng J, Pan G (2016). Abundance, composition and activity of denitrifier communities in metal polluted paddy soils. Sci Rep.

[24] Broman E, Motwani NH, Bonaglia S, Landberg T, Nascimento FJA, Sjöling S (2019). Denitrification responses to increasing cadmium exposure in Baltic Sea sediments. Aquat Toxicol.

[25] Guo Q, Li N, Bing Y, Chen S, Zhang Z, Chang S, Chen Y, Xie S (2018). Denitrifier communities impacted by heavy metal contamination in freshwater sediment. Environ Pollut.

[26] Lindgren JF, Hassellöv I-M, Dahllöf I (2014). PAH effects on meio- and microbial benthic communities strongly depend on bioavailability. Aquat Toxicol.

[27] Tobiasson S, Andersson S (2013) Avgångshastighet för metaller och organiska miljögifter i förorenade sediment från Oskarshamns hamn, 2011–2013. Rapport 2013:9. Linnéuniversitetet, Institutionen för biologi och miljö

[28] Bank A (2005) Preliminär miljö- och hälsoriskbedömning av föroreningar i sediment inom hamnbassängen. Rapport nr Oskarshamns hamn 2004:14. Oskarshamns kommun, 1–30

[29] Fathollahzadeh H, Kaczala F, Bhatnagar A, Hogland W (2015). Significance of environmental dredging on metal mobility from contaminated sediments in the Oskarshamn Harbor, Sweden. Chemosphere.

[30] Oskarshamns Kommun (2016) Saneringen av Oskarshamns Hamnbassäng: Projekt- och erfarenhetsrapport 1996–2015. In: Schultzén, J (ed.), Oskarshamn, 1–96

[31] Jönsson BL (2020) Information om saneringen av Oskarshamns Hamnbassäng: Nyhetsbrev 19 - April 2020. Oskarshamn Kommun

[32] Rämö R, Bonaglia S, Nybom I, Kreutzer A, Witt G, Sobek A, Gunnarsson JS (2022). Sediment remediation using activated carbon: effects of sorbent particle size and resuspension on sequestration of metals and organic contaminants. Environ Toxicol Chem.

[33] Björinger P (2011) Sedimentprovtagning delområde E (Djuphåla). O-hamn 2011:13 NIRAS Environment

[34] Blomqvist S, Ekeroth N, Elmgren R, Hall PO (2015). Long overdue improvement of box corer sampling. Mar Ecol Prog Ser.

[35] Blomqvist M, Leonardsson K (2016) A probability based index for assessment of benthic invertebrates in the Baltic Sea. WATERS report.

[36] Leonardsson K, Blomqvist M, Rosenberg R (2009). Theoretical and practical aspects on benthic quality assessment according to the EU-Water Framework Directive–examples from Swedish waters. Mar Pollut Bull.

[37] Swedish EPA (2000). Environmental quality criteria: coasts and seas. Swedish Environ Protection Agency Report.

[38] Nielsen LP (1992). Denitrification in sediment determined from nitrogen isotope pairing. FEMS Microbiol Lett.

[39] De Brabandere L, Bonaglia S, Kononets MY, Viktorsson L, Stigebrandt A, Thamdrup B, Hall POJ (2015). Oxygenation of an anoxic fjord basin strongly stimulates benthic denitrification and DNRA. Biogeochemistry.

[40] International Organization for Standardization (2006) Soil quality — pretreatment of samples for physico-chemical analysis. ISO 11464:2006.

[41] Swedish Institute for Standards (2016) Water quality - application of inductively coupled plasma mass spectrometry (ICP-MS) - part 2: determination of selected elements including uranium isotopes. vol. SS-EN ISO 17294–2:2016. Swedish Standard

[42] Swedish Institute for Standards (2019) Soil, treated biowaste and sludge - determination of dioxins and furans and dioxin-like polychlorinated biphenyls by gas chromatography with high resolution mass selective detection (HR GC-MS). vol. SS-EN 16190:2019. Swedish Standard, 56

[43] Schaanning MT, Beylich B, Gunnarsson JS, Eek E (2021). Long-term effects of thin layer capping in the Grenland fjords, Norway: reduced uptake of dioxins in passive samplers and sediment-dwelling organisms. Chemosphere.

[44] Valderrama JC (1995) Methods of nutrient analysis. In: Hallegraeff GM, ADaCA (ed.) Manual on harmful marine microalgae. Intergovernmental Oceanographic Commision of UNESCO, Paris, 251–268

[45] Westreich ST, Treiber ML, Mills DA, Korf I, Lemay DG (2018). SAMSA2: a standalone metatranscriptome analysis pipeline. BMC Bioinformatics.

[46] Wood DE, Salzberg SL (2014) Kraken: ultrafast metagenomic sequence classification using exact alignments. Genome Biol 15:1–12 10.1186/gb-2014-15-3-r46 PMC4053813 24580807

[47] Lu J, Breitwieser FP, Thielen P, Salzberg SL (2017). Bracken: estimating species abundance in metagenomics data. PeerJ Computer Science.

[48] Broman E, Bonaglia S, Norkko A, Creer S, Nascimento FJ (2020). High throughput shotgun sequencing of eRNA reveals taxonomic and derived functional shifts across a benthic productivity gradient. Mol Ecol.

[49] Hammer Ø, Harper DAT, Ryan PD (2001). PAST: paleontological statistics software package for education and data analysis. Palaeontol Electron.

[50] R Core Team (2013) R: a language and environment for statistical computing. R Foundation for Statistical Computing, Vienna, Austria. URL http://www.R-project.org/.

[51] Fox J, Weisberg S, Adler D, Bates D, Baud-Bovy G, Ellison S, Firth D, Friendly M, Gorjanc G, Graves S (2012) Package ‘car’. Vienna: R Foundation for Statistical Computing: 16

[52] Yan C, Wang F, Geng H, Liu H, Pu S, Tian Z, Chen H, Zhou B, Yuan R, Yao J (2020). Integrating high-throughput sequencing and metagenome analysis to reveal the characteristic and resistance mechanism of microbial community in metal contaminated sediments. Sci Total Environ.

[53] Harrell Jr FE, Dupont C (2008) Hmisc: Harrell miscellaneous. R package version 3

[54] Robinson MD, McCarthy DJ, Smyth GK (2010). edgeR: a Bioconductor package for differential expression analysis of digital gene expression data. Bioinformatics.

[55] Oskarshamns Kommun (2005) Huvudstudierapport: Sanering av hamnbassängen i Oskarshamn. In: Bank, A, Carlsson, B (eds.), Oskarshamn, 1–77

[56] Van den Berg M, Birnbaum LS, Denison M, De Vito M, Farland W, Feeley M, Fiedler H, Hakansson H, Hanberg A, Haws L (2006). The 2005 World Health Organization reevaluation of human and mammalian toxic equivalency factors for dioxins and dioxin-like compounds. Toxicol Sci.

[57] Deutsch B, Forster S, Wilhelm M, Dippner JW, Voss M (2010). Denitrification in sediments as a major nitrogen sink in the Baltic Sea: an extrapolation using sediment characteristics. Biogeosciences.

[58] Bonaglia S, Deutsch B, Bartoli M, Marchant HK, Brüchert V (2014). Seasonal oxygen, nitrogen and phosphorus benthic cycling along an impacted Baltic Sea estuary: regulation and spatial patterns. Biogeochemistry.

[59] Thamdrup B (2012). New pathways and processes in the global nitrogen cycle. Annu Rev Ecol Evol Syst.

[60] Zárate A, Dorador C, Valdés J, Molina V, Icaza G, Pacheco AS, Castillo A (2021). Benthic microbial diversity trends in response to heavy metals in an oxygen-deficient eutrophic bay of the Humboldt current system offshore the Atacama Desert. Environ Pollut.

[61] Tsai Y-P, You S-J, Pai T-Y, Chen K-W (2005). Effect of cadmium on composition and diversity of bacterial communities in activated sludges. Int Biodeterior Biodegradation.

[62] Prokopenko MG, Hirst MB, De Brabandere L, Lawrence DJP, Berelson WM, Granger J, Chang BX, Dawson S, Crane Iii EJ, Chong L, Thamdrup B, Townsend-Small A, Sigman DM (2013). Nitrogen losses in anoxic marine sediments driven by *Thioploca*–anammox bacterial consortia. Nature.

[63] Zhang N, Chen H, Lyu Y, Wang Y (2019). Nitrogen removal by a metal-resistant bacterium, *Pseudomonas putida* ZN1, capable of heterotrophic nitrification–aerobic denitrification. J Chem Technol Biotechnol.

[64] Raja CE, Anbazhagan K, Selvam GS (2006). Isolation and characterization of a metal-resistant *Pseudomonas Aeruginosa* strain. World J Microbiol Biotechnol.

[65] Carlson CA, Ingraham JL (1983). Comparison of denitrification by *Pseudomonas stutzeri*, *Pseudomonas aeruginosa*, and *Paracoccus denitrificans*. Appl Environ Microbiol.

[66] Thureborn P, Franzetti A, Lundin D, Sjöling S (2016). Reconstructing ecosystem functions of the active microbial community of the Baltic Sea oxygen depleted sediments. PeerJ.

[67] Thureborn P, Lundin D, Plathan J, Poole AM, Sjoberg BM, Sjoling S (2013). A metagenomics transect into the deepest point of the Baltic Sea reveals clear stratification of microbial functional capacities. PLOS One.

[68] Kuypers MMM, Marchant HK, Kartal B (2018). The microbial nitrogen-cycling network. Nat Rev Microbiol.

[69] Bağcı C, Patz S, Huson DH (2021). DIAMOND+MEGAN: fast and easy taxonomic and functional analysis of short and long microbiome sequences. Current Protocols.

[70] Broman E, Sun X, Stranne C, Salgado MG, Bonaglia S, Geibel M, ..., Nascimento FJ (2020) Low abundance of methanotrophs in sediments of shallow boreal coastal zones with high water methane concentrations. Front Microbiol 11:1536 10.3389/fmicb.2020.01536 PMC7362727 32733420

[71] Broman E, Bonaglia S, Holovachov O, Marzocchi U, Hall PO, Nascimento FJ (2020) Uncovering diversity and metabolic spectrum of animals in dead zone sediments. Communications Biology 3:106. 10.1038/s42003-020-0822-7 10.1038/s42003-020-0822-7 PMC7060179 32144383

[72] Broman E, Izabel-Shen D, Rodríguez-Gijón A, Bonaglia S, Garcia SL, Nascimento FJA (2022). Microbial functional genes are driven by gradients in sediment stoichiometry, oxygen, and salinity across the Baltic benthic ecosystem. Microbiome.

[73] Rocca JD, Hall EK, Lennon JT, Evans SE, Waldrop MP, Cotner JB, Nemergut DR, Graham EB, Wallenstein MD (2015). Relationships between protein-encoding gene abundance and corresponding process are commonly assumed yet rarely observed. ISME J.

[74] Magalhães C, Costa J, Teixeira C, Bordalo AA (2007). Impact of trace metals on denitrification in estuarine sediments of the Douro River estuary, Portugal. Mar Chem.

[75] Wittorf L, Roger F, Alsterberg C, Gamfeldt L, Hulth S, Sundbäck K, Jones CM, Hallin S (2020) Habitat diversity and type govern potential nitrogen loss by denitrification in coastal sediments and differences in ecosystem-level diversities of disparate N2O reducing communities. FEMS Microbiol Ecol 96:fiaa091 10.1093/femsec/fiaa091 PMC7428367 32662514

[76] Albert S, Bonaglia S, Stjärnkvist N, Winder M, Thamdrup B, Nascimento FJA (2021). Influence of settling organic matter quantity and quality on benthic nitrogen cycling. Limnol Oceanogr.

[77] Bonaglia S, Nascimento FJA, Bartoli M, Klawonn I, Brüchert V (2014). Meiofauna increases bacterial denitrification in marine sediments. Nat Commun.

[78] Bonaglia S, Rämö R, Marzocchi U, Le Bouille L, Leermakers M, Nascimento FJA, Gunnarsson JS (2019). Capping with activated carbon reduces nutrient fluxes, denitrification and meiofauna in contaminated sediments. Water Res.

[79] Bonaglia S, Bartoli M, Gunnarsson JS, Rahm L, Raymond C, Svensson O, Yekta SS, Brüchert V (2013). Effect of reoxygenation and Marenzelleria spp. bioturbation on Baltic Sea sediment metabolism. Mar Ecol Prog Ser.

[80] Jenkins MC, Kemp WM (1984). The coupling of nitrification and denitrification in two estuarine sediments 1, 2. Limnol Oceanogr.

[81] Shiang FuQ, Barkovskii AL, Adriaens P (2005). Microbial dechlorination of dioxins in estuarine enrichment cultures: effects of respiratory conditions and priming compound on community structure and dechlorination patterns. Mar Environ Res.

[82] Holliger C, Wohlfarth G, Diekert G (1998). Reductive dechlorination in the energy metabolism of anaerobic bacteria. FEMS Microbiol Rev.

[83] Zanaroli G, Negroni A, Häggblom MM, Fava F (2015). Microbial dehalogenation of organohalides in marine and estuarine environments. Curr Opin Biotechnol.

[84] Bunge M, Lechner U (2009). Anaerobic reductive dehalogenation of polychlorinated dioxins. Appl Microbiol Biotechnol.

[85] Gruden CL, Fu QS, Barkovskii AL, Albrecht ID, Lynam MM, Adriaens P, Häggblom MM, Bossert ID (2003). Dechlorination of sediment dioxins: catalysts, mechanisms, and implications for remedial strategies and dioxin cycling. Dehalogenation: microbial processes and environmental applications.

[86] Braker G, Zhou J, Wu L, Devol AH, Tiedje JM (2000). Nitrite reductase genes (nirK and nirS) as functional markers to investigate diversity of denitrifying bacteria in pacific northwest marine sediment communities. Appl Environ Microbiol.

[87] Throbäck IN, Enwall K, Jarvis Å, Hallin S (2004). Reassessing PCR primers targeting nirS, nirK and nosZ genes for community surveys of denitrifying bacteria with DGGE. FEMS Microbiol Ecol.

