# Supplementary material for: Long-Term Pollution Does Not Inhibit Denitrification and DNRA by Adapted Benthic Microbial Communities
Source: Microb Ecol. 2023 May 24;86(4):2357–72. doi: 10.1007/s00248-023-02241-7 (PMC10640501; doi:10.1007/s00248-023-02241-7)
Supplement: Supplementary file 7 — Supplementary file7 (DOCX 2.55 MB) [file 248_2023_2241_MOESM7_ESM.docx]

**Long-term pollution does not significantly inhibit denitrification and DNRA by adapted benthic microbial communities**

Elias Broman, Mohanad Abdelgadir, Stefano Bonaglia, Sara C. Forsberg, Johan Wikström, Jonas S. Gunnarsson, Francisco J.A. Nascimento, and Sara Sjöling

**Supplementary Information**

*Captions to supplementary data files*

**Data S1** The data file shows sequence facility ids, sample names, number of read-pairs yielded after sequencing, quality trimming and merging details, number of 16S rRNA gene sequences classified against SILVA, and meagenome sequences classified with DIAMOND against the MEGAN supplied KEGG database.

**Data S2** The data file shows the denitrification and DNRA results from the IPT experiment.

**Data S3** The data file shows: sheet 1) KEGG classifications in the nitrogen metabolism pathway, and sheet 2) all the KEGG classifications for all pathways detected in th samples. The table shows normalized reads counts derived from the MEGAN software normalized read counts function (sub-sample to lowest sample size).

**Data S4** The data file shows MEGAN taxonomy linked to KEGG classifications (metal related genes, Dioxin pathway, and Nitrogen metabolism pathways). The results are based on linking taxonomy and KEGG classifications having the same read name. The table shows normalized counts as counts per million (CPM) values.

**Data S5** The data file shows the edgeR results (only results with *FDR* < 0.05 are reported). The table shows log fold-change values based on pairwise comparisons between stations.

**Data S6** The data file shows 16S rRNA gene sequences (sheet 1) extracted from the metagenome (using SortMeRNA) and classified against the SILVA database. The table shows raw read counts.

*Supplementary figures*


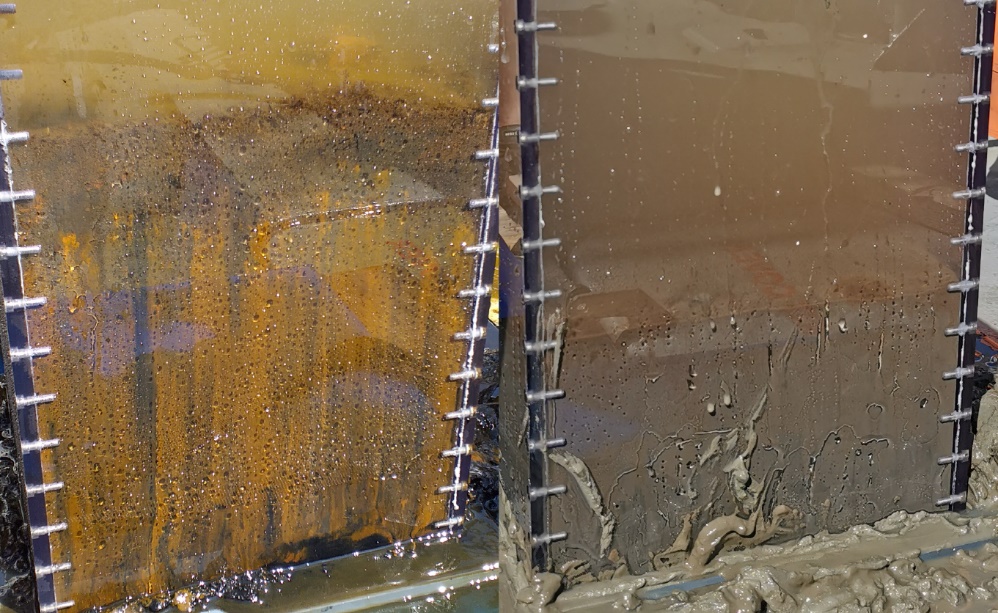


**Figure S1** Polluted sediment sampled in the harbor of Oskarshamn (station OSK 1, *left photo*), and sediment sampled from Askö (station ASK, *right photo*).


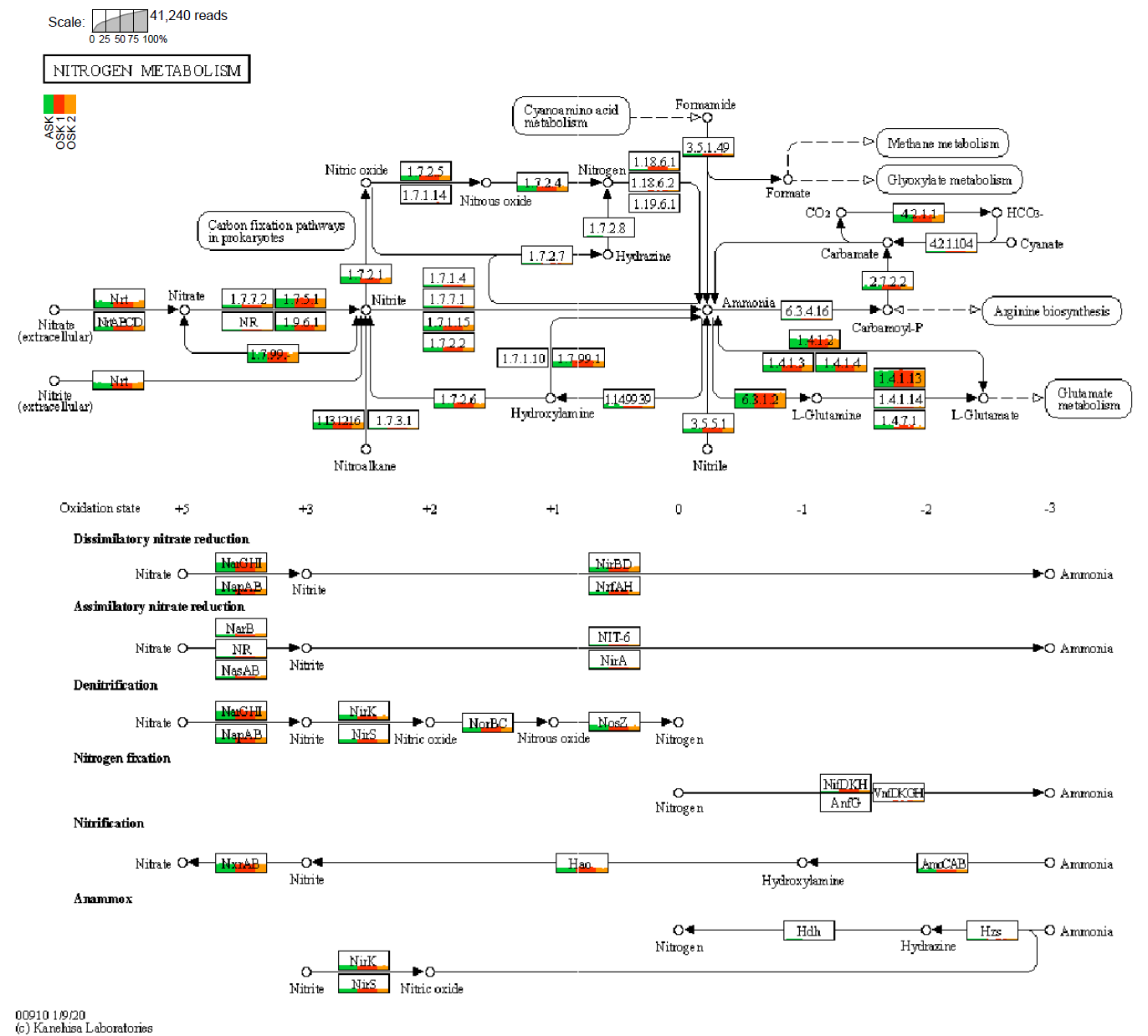


**Figure S2** The KEGG Nitrogen metabolism pathway map with the number of normalized read counts indicated in each cell. The colored bars inside the cells denote the different stations, with green bars as ASK (*n* = 7), red OSK 1 (*n* = 7), and orange OSK 2 (*n* = 4). Note that the bars are based on a square root scale (top left corner), and that the top each cell represents the highest number or reads (41,240) detected in a single cell (glutamate synthase (NADPH) enzyme EC 1.4.1.13). The top of the map shows the enzymes (with a following EC number) in each pathway, while the lower part of the map gives an overview of the pathways and the specific gene names for each step. The arrows denote the direction of the metabolic process.

**
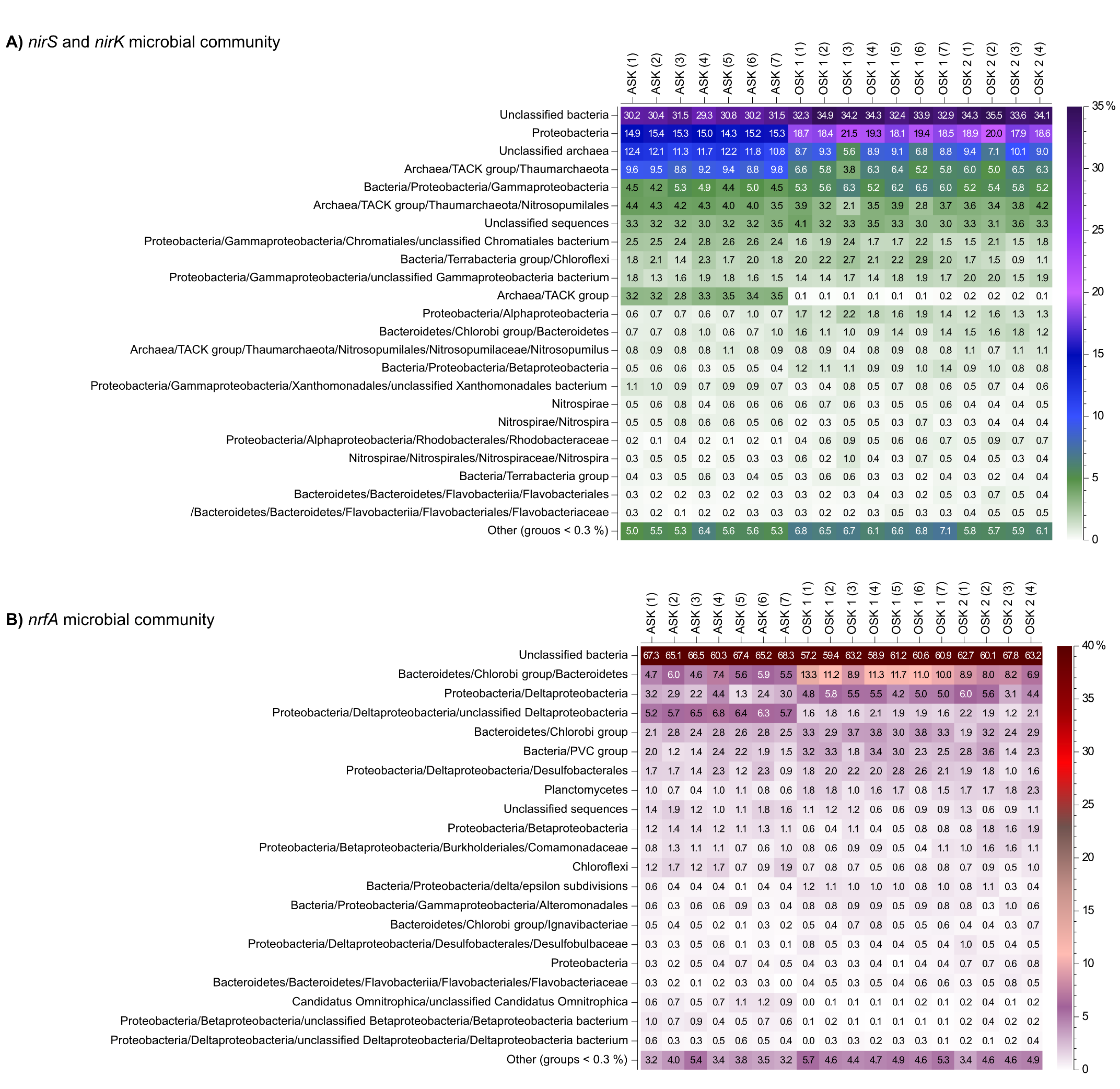
**

**Figure S3** Heatmap showing the lowest classified taxonomy of reads classified to genes A) *nirS* and *nirK*, and B) *nrfA*. Other denotes taxonomic groups < 0.3 % (average of all samples). The values show relative abundance (%) for each community.

**
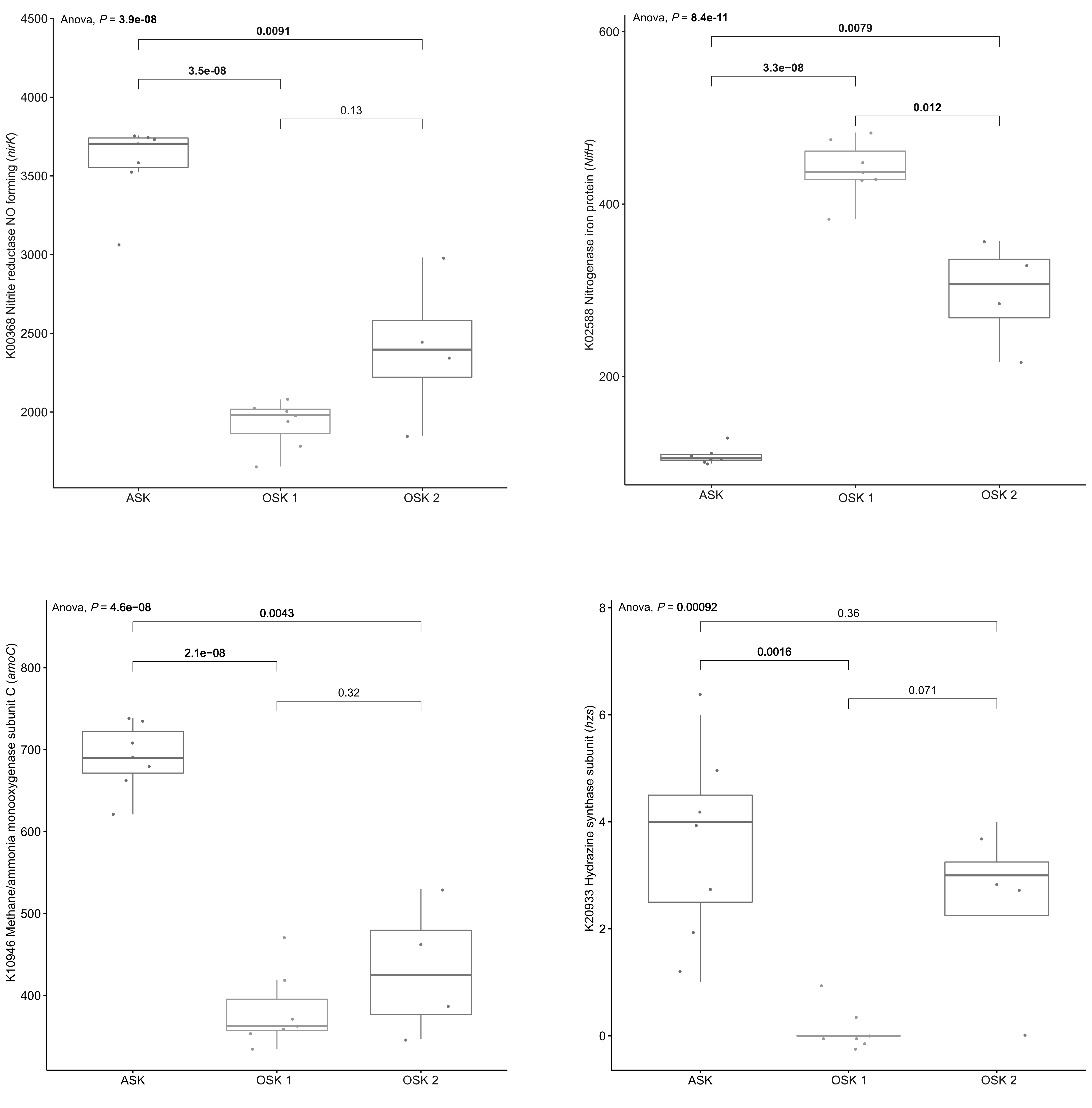
**

**
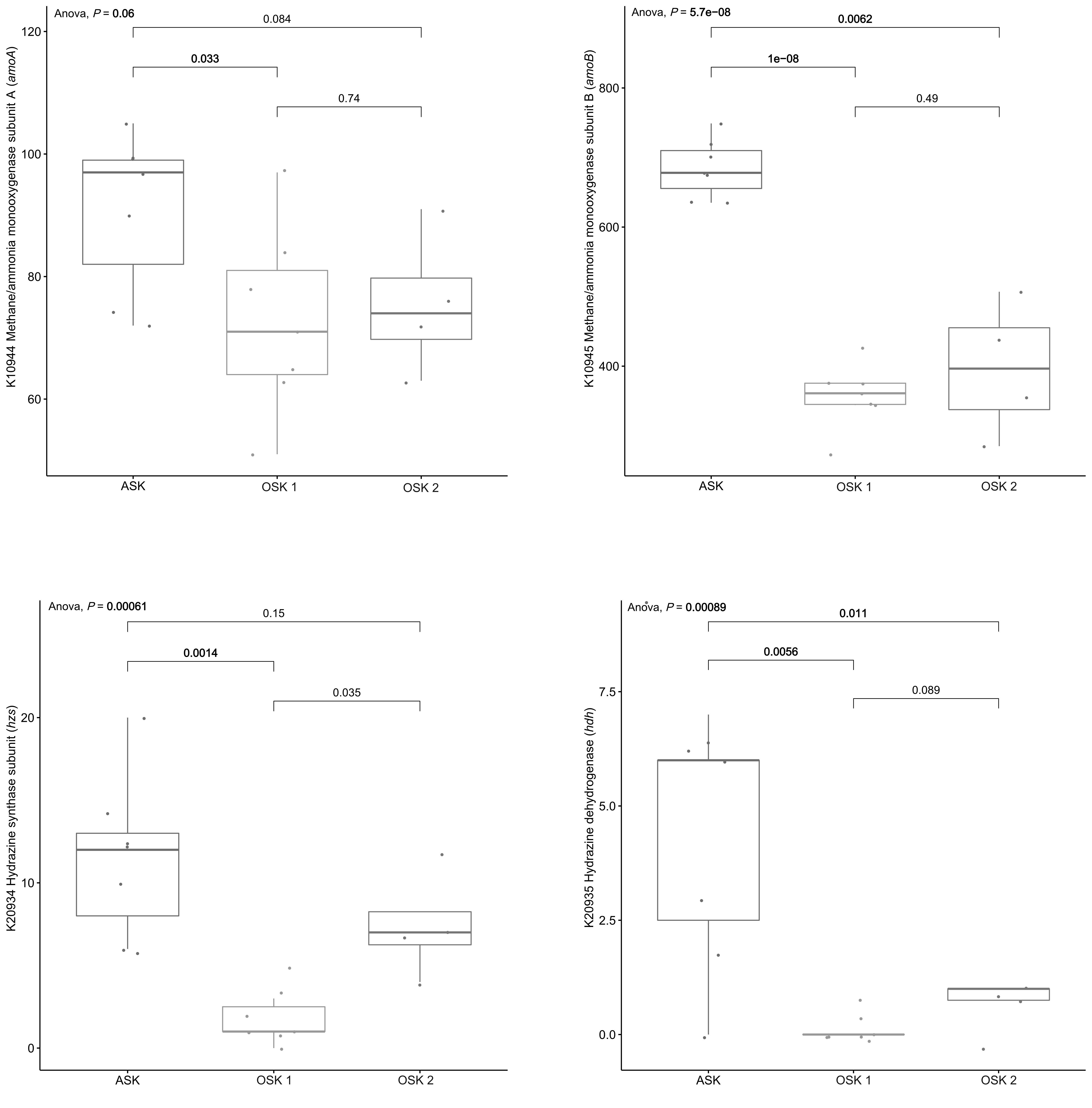
**

**Figure S4** Boxplots showing selected KEGG KO identifiers and their associated gene names. The data is based on normalized read counts, and the One-Way ANOVA results show the results for the whole model (including all three stations), while the p-value between stations are based on pairwise t-tests. P-values < 0.05 have been marked with bold text. Note that two subunits are shown for *hzs* (KEGG KO K20933 and K20934).

**Supplementary methods**

*Denitrification and DNRA rate measurements (IPT incubation)*

The water level inside the incubation chamber was lowered so the cores were no longer submersed, and three water samples were collected into 15 mL centrifuge tubes (Sarstedt) and stored at -20°C. These samples were later used to determine NO_x_^-^ (NO_2_^-^+NO_3_^-^) concentrations in the initial bottom water. The cores were then spiked with a 10 mM Na^15^NO_3_ solution (99.4 ^15^N atom %; Sigma-Aldrich, DE) to a final concentration of 20 µM ^15^NO_3_^-^ (based on calculated water volume inside each core). This was followed by 1.5-hour pre-incubation necessary for the added ^15^N to homogenize with the *in situ* ^14^N inside the cores (De Brabandere et al., 2015). After 20–30 minutes of pre-incubation, the water inside three random cores was subsampled into 15 mL centrifuge tubes and stored at -20°C. These samples were used to determine NO_x_^-^ concentrations after having added the ^15^N labelled NO_3_^‑^. After pre-incubation one core was removed from the incubation chamber and subsampled for N_2_ analysis as described below. The other 9 cores were capped without air-bubbles trapped inside, stirred using magnetic bars as described above, and incubated for 6 hours. At the end of the incubation the cores were handled one at a time. First the core was uncapped and the top 2 cm sediment was lightly mixed with the overlying bottom water to create a slurry (sediment + water). After 1–2 minutes ~20 mL of water inside the core was subsampled for N_2_ analysis (*n* = 9 per station), with a syringe attached to a transparent PVC tube, into a 12 mL Exetainer® vial (Labco, UK) avoiding air intrusion. This sample was preserved with 250 μL 6 M ZnCl_2_ to stop biological activity, stored upside down, and kept refrigerated until further analysis. A similar sample was taken but was filtered through a 0.45 µm filter (Filtropur S, Sarstedt) for analysis of dissolved ^15^NH_4_^+^ (*n* = 9 per station). These samples were also preserved with 250 μL 6 M ZnCl_2_, stored upside down, and kept refrigerated until further analysis. *In situ* DNRA rates were corrected for adsorption of the produced ^15^NH_4_^+^ to the sediment particles, for which a loss of 50 % was assumed, according to Laima (1994) and De Brabandere et al. (2015). The N_2_ samples were analyzed with a gas chromatographer isotope ratio mass spectrometer (GC-IRMS) at UC Davis facilities, University of California. One sample from OSK 1 for N_2_ analysis was damaged during transportation and was therefore excluded in the final dataset. The NH_4_^+^ samples were oxidized to N_2_ by the addition of alkaline hypobromite (Warembourg, 1993) and then analyzed using GC-IRMS at the University of Southern Denmark. Denitrification and DNRA rates were determined by calculating excesses of ^29^N_2_, ^30^N_2_ and ^15^NH_4_^+^, and the actual denitrification/DNRA rates (D14, DNRA) and denitrification of NO_3_^-^ produced by nitrification (D14N) are reported (Bonaglia et al., 2017; De Brabandere et al., 2015; Nielsen, 1992).

*Detailed bioinformatics*

Illumina TruSeq adapters were removed from the sequences using SeqPrep 1.2 (St John, 2011), followed by removal of any leftover PhiX control sequences by mapping sequence reads against the PhiX-174 genome (NCBI accession: NC_001422) using bowtie2 2.3.5.1 (Langmead and Salzberg, 2012). The data was quality trimmed with Trimmomatic 0.36 (Bolger et al., 2014) using settings: LEADING:20 TRAILING:20 MINLEN:100. The final quality trimmed data was controlled using FastQC 0.11.9 (Andrews, 2010) and MultiQC 1.9 (Ewels et al., 2016), and had on average 104.1 million sequences (min: 80.4, max: 131.8), 150 length (bp), and a Phred33 quality score of 36 per base. See Data S1 for full quality trimming details per sample.

The functional gene analysis closely followed the SAMSA2 pipeline (Westreich et al., 2018). In more detail, the paired without unpaired (PwU) quality trimmed R1 and R2 reads from trimmomatic were merged using PEAR 0.9.10 (Zhang et al., 2014) using default settings. The merged reads (ca 20% of the data) were combined with the notCombined forward reads as recommended by the SAMSA2 pipeline (Westreich et al., 2018). This was followed by classification of the reads against the NCBI NR database (database downloaded: May 30 2020) using DIAMOND 2.0.4 (Buchfink et al., 2015) with default settings (i.e. an e-value threshold of 0.001, up to 25 matches per query, and tantan repeat masking algorithm to remove spurious hits (Buchfink et al., 2015; Frith, 2011). The .daa files were meganized to retrieve NCBI taxonomy and KEGG functional annotations using the database “megan-map-Jan2021-ue.db” with the tool “daa-meganizer” using default settings, both supplied with MEGAN6 Ultimate Edition (Huson et al., 2007). This includes an additional filtering step per read query where classifications < 50 blast bit score are removed, as well as excluding hits per read query outside the top 10 % of the highest bit score. On average 59,309,518 reads (min: 45,201,360, max: 74,286,032) had been classified with DIAMOND against the NCBI NR database and imported into MEGAN. The meganized files were normalized for read counts between samples using the “compute-comparison” tool supplied with MEGAN. This process sub-samples to the lowest sample size (45.2 million sequences with an average of 19.8 million that could be linked to KEGG KO identifiers with a KEGG PATHWAY; Data S1) and combined the meganized .daa files to one combined dataset. The final data was then analyzed with MEGAN6 Ultimate Edition to visualize KEGG gene annotations, KEGG pathway maps, and extract data for statistical analysis. The MEGAN taxonomy and KEGG classifications were linked by extracting read names alongside taxonomy and functional classifications using the daa2info tool supplied with MEGAN. Information from exact matching read names were then linked and the counts data were normalized as counts per million values (CPM, i.e. relative proportion × 1 000 000).

The taxonomic annotation closely followed the procedure described by Broman et al. (2020). In more detail, the quality trimmed PwU R1 and R2 reads were loaded into SortMeRNA 4.2.0 (Kopylova et al., 2012) (settings: --paired_out --num_alignments 1) with the software supplied databases to extract SSU rRNA gene sequences. The 16S rRNA gene sequences were taxonomically classified using Kraken 2.1.0 (Wood et al., 2019) with a paired-end setup (parameter: --paired) against the SILVA (database downloaded: August 1 2020). To estimate relative abundances on genera level the software Bracken 2.6.0 (Lu et al., 2017; Lu and Salzberg, 2020) was used with the Kraken2 reports and the settings: -r 150 -l G -t 10 (denoting a read length of 150, classified to genus level, and a minimum threshold of 10 counts for a genus to be present). The bracken reports for each sample were combined into a biom-format file using the python package kraken-biom 1.0.1 (parameters: ---fmt hdf5 -max D --min G) (Dabdoub, 2016), which was further converted to a tab delimited table using the python package biom-format 2.1.7 (McDonald et al., 2012). The final data consisted of, on average, 43,024 (min: 30,819, max: 66,042) 16S rRNA gene sequences per sample classified as bacteria and archaea. The final data was imported and analyzed in the software Explicet 2.10.5 (Robertson et al., 2013) and the data was normalized as relative abundances (%) between samples.

**Supplementary results and discussion**

*Difference in N_2_-fixation, ammonia oxidation, and anammox genes*

Since the nitrogen fixation capacity has been shown to be overrepresented in deep water sediments in the Baltic Sea compared with several sediments globally (Thureborn et al., 2013), likely reflecting the general eutrophied conditions, we included analyses of the nitrogen fixation pathway in this study. We observed a significant overrepresentation of the nitrogenase coding *nifH* in the polluted harbor sediments compared with the reference station. At OSK 1 the read count was ~1.5 times higher than at OSK 2 and ~4 times higher than that of ASK (student’s t-test, *P* < 0.05; Fig. S3). The MEGAN taxonomy indicated that the differences in *nifH* genes could mainly be attributed to Desulfuromonadales at OSK 1 (One-Way ANOVA test, *F* = 27, *P* < 0.01; Data S4). In addition to *nifH* the other nitrogenase encoding genes *nifD* and *nifK* were also attributed to Desulfuromonadales (Data S4). There was also a difference in ammonia oxidation genes among stations, however, *amoA* was relatively more abundant at the reference station ASK compared with OSK 1, whereas *amoB* plus *amoC* were more abundant at ASK compared with both OSK 1 and OSK 2 (One-Way ANOVA with pairwise student’s t-tests, *P* < 0.05; Data S3). Anammox hydrazine synthase and dehydrogenase genes (*hzs* and *hdh*, respectively) were also detected but at low read counts (< 30 reads per sample, and only one read for *hdh* at OSK 1; Fig. S3).

We also observed more N_2_-fixation *nifH* genes at the outer harbor stations when compared with ASK. Nitrogen fixation capacity in the sediments is not unexpected as previous research has demonstrated complete genetic prerequisites for nitrogen fixation even at deep water sediments of the Baltic Sea (Thureborn et al., 2016). Many of the *nifH* genes could be attributed to Desulfuromonadales, that was also prominent in the metal gene data, and experimentally have been shown to likely assimilate N_2_ (Kapili et al., 2020). Interestingly, some bacterial members in this taxonomic order are known to carry *nrfA* genes (Bu et al., 2017) and have also been described to be important for DNRA (Tang et al., 2020). Our data show that the capacity for N_2_-fixation was higher at the harbor stations, and further suggest that some of these bacteria that carry N_2_-fixation genes might have been conducting DNRA.

**References**

Andrews, S. 2010. FastQC: a quality control tool for high throughput sequence data.

Bolger, A.M., Lohse, M. and Usadel, B. 2014. Trimmomatic: A flexible trimmer for Illumina sequence data. Bioinformatics 30(15), 2114–2120.

Bonaglia, S., Hylén, A., Rattray, J.E., Kononets, M.Y., Ekeroth, N., Roos, P., Thamdrup, B., Brüchert, V. and Hall, P.O.J. 2017. The fate of fixed nitrogen in marine sediments with low organic loading: an in situ study. Biogeosciences 14(2), 285-300.

Broman, E., Bonaglia, S., Norkko, A., Creer, S. and Nascimento, F.J. 2020. High throughput shotgun sequencing of eRNA reveals taxonomic and derived functional shifts across a benthic productivity gradient. Molecular Ecology 30(13), 3023-3039.

Bu, C., Wang, Y., Ge, C., Ahmad, H.A., Gao, B. and Ni, S.-Q. 2017. Dissimilatory Nitrate Reduction to Ammonium in the Yellow River Estuary: Rates, Abundance, and Community Diversity. Sci Rep. 7(1), 6830.

Buchfink, B., Xie, C. and Huson, D.H. 2015. Fast and sensitive protein alignment using DIAMOND. Nat Meth 12(1), 59-60.

Dabdoub, S. 2016 kraken-biom, <https://github.com/smdabdoub/kraken-biom>.

De Brabandere, L., Bonaglia, S., Kononets, M.Y., Viktorsson, L., Stigebrandt, A., Thamdrup, B. and Hall, P.O.J. 2015. Oxygenation of an anoxic fjord basin strongly stimulates benthic denitrification and DNRA. Biogeochemistry 126(1), 131-152.

Ewels, P., Magnusson, M., Käller, M. and Lundin, S. 2016. MultiQC: summarize analysis results for multiple tools and samples in a single report. Bioinformatics 32(19), 3047-3048.

Frith, M.C. 2011. Gentle Masking of Low-Complexity Sequences Improves Homology Search. PLOS ONE 6(12), e28819.

Huson, D.H., Auch, A.F., Qi, J. and Schuster, S.C. 2007. MEGAN analysis of metagenomic data. Genome research 17(3), 377-386.

Kapili, B.J., Barnett, S.E., Buckley, D.H. and Dekas, A.E. 2020. Evidence for phylogenetically and catabolically diverse active diazotrophs in deep-sea sediment. The ISME Journal 14(4), 971-983.

Kopylova, E., Noé, L. and Touzet, H. 2012. SortMeRNA: fast and accurate filtering of ribosomal RNAs in metatranscriptomic data. Bioinformatics 28(24), 3211-3217.

Laima, M.C.J. 1994. Is KCl a reliable extractant of15NH4+added to coastal marine sediments? Biogeochemistry 27(2), 83-95.

Langmead, B. and Salzberg, S.L. 2012. Fast gapped-read alignment with Bowtie 2. Nat Meth 9, 357.

Lu, J., Breitwieser, F.P., Thielen, P. and Salzberg, S.L. 2017. Bracken: estimating species abundance in metagenomics data. PeerJ Computer Science 3, e104.

Lu, J. and Salzberg, S.L. 2020. Ultrafast and accurate 16S rRNA microbial community analysis using Kraken 2. Microbiome 8(1), 124.

McDonald, D., Clemente, J.C., Kuczynski, J., Rideout, J.R., Stombaugh, J., Wendel, D., Wilke, A., Huse, S., Hufnagle, J. and Meyer, F. 2012. The Biological Observation Matrix (BIOM) format or: how I learned to stop worrying and love the ome-ome. GigaScience 1(1), 7.

Nielsen, L.P. 1992. Denitrification in sediment determined from nitrogen isotope pairing. FEMS Microbiology Letters 86(4), 357-362.

Robertson, C.E., Harris, J.K., Wagner, B.D., Granger, D., Browne, K., Tatem, B., Feazel, L.M., Park, K., Pace, N.R. and Frank, D.N. 2013. Explicet: graphical user interface software for metadata-driven management, analysis and visualization of microbiome data. Bioinformatics 29(23), 3100-3101.

St John, J. 2011 SeqPrep. <https://github.com/jstjohn/SeqPrep>.

Tang, S., Liao, Y., Xu, Y., Dang, Z., Zhu, X. and Ji, G. 2020. Microbial coupling mechanisms of nitrogen removal in constructed wetlands: A review. Bioresource Technology 314, 123759.

Thureborn, P., Franzetti, A., Lundin, D. and Sjöling, S. 2016. Reconstructing ecosystem functions of the active microbial community of the Baltic Sea oxygen depleted sediments. PeerJ 4, e1593.

Thureborn, P., Lundin, D., Plathan, J., Poole, A.M., Sjoberg, B.M. and Sjoling, S. 2013. A metagenomics transect into the deepest point of the Baltic Sea reveals clear stratification of microbial functional capacities. PLOS One 8(9), e74983.

Warembourg, F.R. 1993. Nitrogen fixation in soil and plant systems. Nitrogen isotope techniques, 127-156.

Westreich, S.T., Treiber, M.L., Mills, D.A., Korf, I. and Lemay, D.G. 2018. SAMSA2: a standalone metatranscriptome analysis pipeline. BMC bioinformatics 19(1), 175-175.

Wood, D.E., Lu, J. and Langmead, B. 2019. Improved metagenomic analysis with Kraken 2. Genome Biol 20(1), 257.

Zhang, J., Kobert, K., Flouri, T. and Stamatakis, A. 2014. PEAR: a fast and accurate Illumina Paired-End reAd mergeR. Bioinformatics 30(5), 614-620.
